# When Virchow's law fails: Crouzon syndrome as a test case for quantitative suture closure–endocranial shape relationships

**DOI:** 10.1111/joa.70237

**Published:** 2026-09-14

**Authors:** F. W. R. Steup, E. V. Groenenboom, O. Delassus, A. Balzeau, A. Profico, G. Paternoster, M. Moazen, R. H. Khonsari, L. S. Van De Lande, M. Taverne

**Affiliations:** ^1^ Department of Oral and Maxillofacial Surgery Erasmus University Medical Center Rotterdam The Netherlands; ^2^ Craniofacial Growth and Form Lab Imagine Institute, UMR 1163 Paris France; ^3^ PaleoFED Team, UMR 7194, CNRS, Département Homme et Environnement Muséum National d'Histoire Naturelle, Musée de l'Homme Paris France; ^4^ Department of Biology University of Pisa Pisa Italy; ^5^ Department of Neurosurgery Necker – Enfants Malades University Hospital Paris France; ^6^ Department of Mechanical Engineering University College London London UK; ^7^ Department of Maxillofacial Surgery and Plastic Surgery Necker ‐ Enfants Malades University Hospital Paris France

**Keywords:** brachycephaly, craniosynostosis, *FGFR2*, geometric morphometrics, intracranial cavity, sutures

## Abstract

Crouzon syndrome is a syndromic craniosynostosis characterized by premature fusion of cranial sutures and variability in cranial phenotype, which is poorly explained by classical growth rules such as Virchow's law. This study tested whether quantitative, continuous assessment of calvarial suture patency can help explain endocranial shape variability when categorical suture descriptions fail. We retrospectively analyzed preoperative cranial CT scans from children with FGFR2‐related bicoronal craniosynostosis and age‐matched controls (birth to 4 years). Endocasts were reconstructed using a semiautomated pipeline and analyzed with three‐dimensional geometric morphometrics. Suture patency of major calvarial sutures was quantified as a continuous measure and converted into age‐adjusted residuals, allowing deviations from control closure dynamics to be assessed independently of age. Intracranial volume increased with age at comparable rates in both groups, indicating preserved global endocranial growth. However, endocranial shape and its allometric relationship to growth differed between groups. While age‐adjusted suture patency showed only weak associations with shape in controls, suture residuals covaried strongly with coherent, system‐level endocast shape changes in Crouzon patients, including alterations in anteroposterior proportions and cranial fossa configuration. Morphological clusters did not map one‐to‐one onto suture closure patterns, indicating nonlocal and context‐dependent effects. These findings suggest that suture‐based *models* such as Virchow's law fail to predict cranial shape in syndromic craniosynostosis. By treating suture patency as a continuous, age‐adjusted variable embedded within a coupled brain–skull growth system, this study suggests that Crouzon syndrome could act as a test case for quantitative, system‐level models of craniofacial development.

## Graphical abstract

Crouzon syndrome shows preserved intracranial volume but profoundly altered endocranial shape, with disrupted allometric coupling relative to controls. Treating calvarial suture patency as a continuous, age‐adjusted variable reveals strong system‐level covariation with endocranial morphology, effects invisible to conventional categorical suture scoring. These findings demonstrate why Virchow's law fails in syndromic craniosynostosis and support a coupled brain–skull growth model.

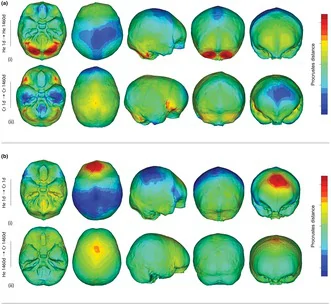

## 1 INTRODUCTION

Craniosynostosis encompasses a heterogeneous group of congenital craniofacial disorders characterized by the premature fusion of one or more cranial vault sutures, leading to altered skull growth and, in some cases, secondary effects on intracranial volume (ICV) and brain development. Among syndromic forms, Crouzon syndrome is caused by gain‐of‐function mutations in the fibroblast growth factor receptor 2 (*FGFR2*) gene. It is most frequently associated with premature bicoronal suture fusion, with variable involvements of the sagittal, metopic, and lambdoid sutures (Johnson & Wilkie, 2011; Renier et al., 2000). Despite its relatively homogeneous genetic basis, cranial and craniofacial phenotypes in Crouzon syndrome are remarkably heterogeneous, even among individuals carrying identical *FGFR2* mutations (Twigg & Wilkie, 2015a). However, recent evidence has shown there might be a relation between protein variant and phenotype (Hennocq et al., 2024). The genotype–phenotype correlation remains a major challenge considering clinical, developmental, and biomechanical perspectives (Morriss‐Kay & Wilkie, 2005; Richtsmeier & Flaherty, 2013).

Premature suture fusion disrupts normal skull growth and is associated with a wide range of complications, including intracranial hypertension and Chiari malformation (Bannink et al., 2010; Massimi et al., 2019; Sawh‐Martinez & Steinbacher, 2019; Fearon et al., 2022). Intracranial hypertension is observed in up to 62.5% of patients with Crouzon syndrome (Renier et al., 2000) and has been linked to reduced cortical thickness, optical nerve atrophy, and altered cognitive development (Wilson et al., 2020). However, the total ICV is often preserved in syndromic craniosynostosis and does not reliably predict intracranial pressure, either before or after surgical intervention (Fok et al., 1992; Langvatn et al., 2019; Tovetjärn et al., 2014). These findings support the growing view that neurological risk is more closely related to regional deformation of the cranial cavity and altered brain–skull interactions than to global volume restriction (Barbeito‐Andrés et al., 2020; Geoffroy et al., 2022; Lieberman et al., 2000).

Cranial sutures are now understood as dynamic, mechanosensitive growth interfaces that integrate genetic regulation, dura mater signaling, and mechanical forces generated by brain growth (Greenwald et al., 2000; Richtsmeier & Flaherty, 2013). Sutural patency therefore represents a temporal and spatial variable process rather than a discrete state, with substantial interindividual variability in timing, extent, and symmetry, particularly in syndromic craniosynostosis (Eswarakumar et al., 2002; Opperman, 2000; Twigg & Wilkie, 2015b; Wang et al., 2021). Yet, suture patency is still most often described categorically (e.g., fused, patent, partially fused) in clinical and morphometric studies, obscuring continuous variation and biochemical factors that may be critical for understanding cranial and endocranial shape outcomes (Twigg & Wilkie, 2015b).

Classical interpretations of craniosynostosis derive from Virchow's law, which describes growth restriction perpendicular to a fused suture, with compensatory growth parallel to the fused suture. While this principle remains a useful first‐order model, it is now widely recognized as insufficiently predictive, particularly in syndromic and multisuture craniosynostoses (Greenwald et al., 2000; Twigg & Wilkie, 2015b). Virchow's law does not account for partial or progressive fusion, biochemical factors, temporal dynamics of fusion, or the active role of brain‐driven forces and intracranial pressure gradients in shaping the cranial vault (Morriss‐Kay & Wilkie, 2005; Richtsmeier et al., 2006). As a result, cranial morphology in syndromic craniosynostosis frequently contradicts suture‐specific growth predictions, suggesting that shape emerges from the interaction of multiple constraints rather than from isolated suture effects alone (Staal et al., 2015).

In this context, endocast morphology provides a particularly informative proxy for intracranial form. Defined by the inner surface of the calvarium and cranial base, the endocast reflects the integrated outcome of brain expansion, sutural constraint, and compensatory skull growth (Richtsmeier & Flaherty, 2013; Sakamoto et al., 2024). Although geometric morphometric studies have documented altered endocranial shapes and developmental trajectories in craniosynostosis (Heuzé et al., 2010; Staal et al., 2015), direct and consistent links between suture closure patterns and endocast shape remain elusive.

Despite advances in three‐dimensional imaging and morphometrics (Gunz et al., 2005; Klingenberg, 2009, 2013, 2016), there is currently no standard framework for incorporating continuous measures of suture patency into predictive models of craniosynostoses (Richtsmeier & Flaherty, 2013; Twigg & Wilkie, 2015b). Treating suture patency as an age‐adjusted, continuous variable may offer a means to separate pathological deviations from normal closure (rather than suture physiological fusion, see Vu et al., 2021) and to test whether graded differences in patency contribute meaningfully to cranial and endocranial shape variation.

Crouzon syndrome, commonly caused by *FGFR2* mutations, presents a large interindividual morphological variability and postnatal progressive suture fusion. Thereby, the syndrome serves as a suitable model to test partial suture patency as a continuous variable in relation to endocast shape variability. The aim of the study was to combine endocast shape analysis with quantitative, continuous assessment of calvarial suture patency to investigate their covariation in patients with *FGFR2*‐related bicoronal craniosynostosis from birth to 4 years of age compared with controls. By integrating three‐dimensional geometric morphometrics and multivariate statistical analyses, we aim to reassess classical growth theories and refine genotype–phenotype interpretation in syndromic craniosynostosis.

We hypothesized that in Crouzon syndrome, classical first‐order suture‐based rules would be insufficient to explain endocranial shape variability, and that continuous, age‐adjusted suture patency would reveal stronger and more structured associations with endocast morphology. We further expected preserved ICV growth but altered size–shape coupling, and a nonexclusive mapping between closure patterns and morphological subgroups, consistent with system‐level growth compensation.

## 2 MATERIALS AND METHODS

### 2.1 Study design and population

This retrospective cross‐sectional study was based on cranial computed tomography (CT) scans extracted from the Picture Archiving and Communication System (PACS) of Hôpital Necker – Enfants malades (AP‐HP, Paris, France), available up to December 21, 2023. The study design specifically aimed to capture early postnatal cranial growth, when both endocranial expansion and sutural remodeling are considered to be most pronounced. Patients were eligible for inclusion if they were clinically diagnosed with Crouzon syndrome and had at least one preoperative CT scan acquired before 4 years of age. To reduce heterogeneity related to marked asymmetry and to focus on a well‐defined synostotic pattern, only individuals presenting with at least partial bicoronal craniosynostosis were included. Patients with cloverleaf skull deformity—defined phenotypically by extreme temporal bulging resulting in a trilobed frontal configuration—were excluded (Machado et al., 2011), as their extreme shape would have driven the main of the observed variance. A control group consisting of children without craniofacial anomalies, selected from the same database, were included. Indications for CT imaging in controls were unrelated to craniofacial anomalies (e.g., trauma, seizures, or suspected infection), and the absence of any malformation was confirmed by two senior craniofacial surgeons (co‐authors RHK, GP). To sample growth evenly through early development, scans were selected across age classes with a denser sampling during infancy and progressively sparser with age (0–12 months: 3 scans per month; 12–24 months: 2 scans per month; 24–48 months: 1 scan per month). One scan per individual was included. When multiple eligible preoperative scans were available for a given patient, the scan was selected based on age requirements for completion of the growth model. In total, 53 individuals with Crouzon syndrome (49.1% female) and 56 controls (50% female) were included. Standardized mean difference (SMD = (μCrouzon – μControls)/SDpooled) showed that age‐matching between Crouzon patients and Controls were near‐perfect match between age distributions (SMD = 0.02). Slice thickness ranged from 0.63 to 1.25 mm (mean 0.68 mm) (Appendix A). In accordance with French regulations, no additional informed consent or ethical approval was required, as the study was retrospective and did not modify clinical management (Simon & Khonsari, 2023).

### 2.2 CT acquisition and preprocessing

**FIGURE 1:**
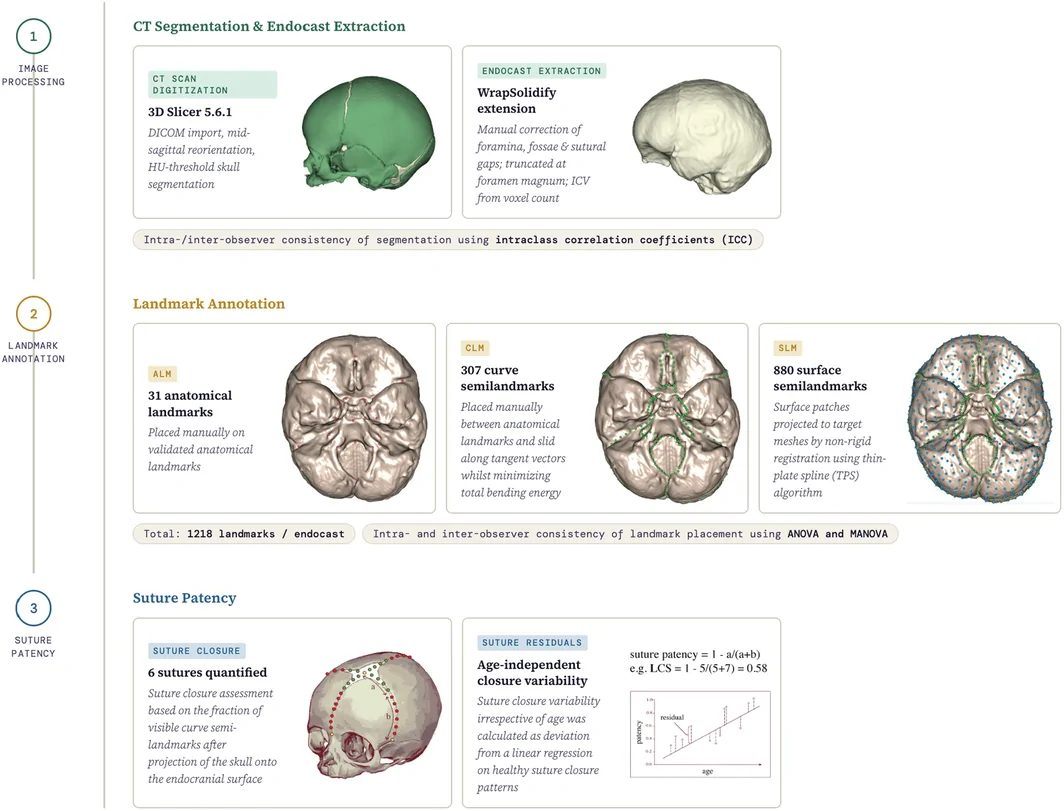
Analytical workflow overview of three methodological steps. CT scan digitization and endocast extraction (Step 1), landmark annotation comprising 1218 landmarks per endocast (Step 2), and quantification of suture patency and age‐independent suture residuals (Step 3).

Only CT scans depicting the full cranium and meeting predefined quality criteria were retained. These criteria included a slice thickness below 1.5 mm and the absence of major motion or metal artifacts. All DICOM files were imported into 3D Slicer 5.6.1 for processing (Fedorov et al., 2012). Volumes were visually inspected and, when necessary, reoriented to ensure approximate alignment of the mid‐sagittal plane prior to segmentation. A graphic overview of the workflow for image processing, landmark annotation, and suture patency calculation is provided (Figure 1).

### 2.3 Endocast segmentation and intracranial volume

Endocast segmentation was performed independently by two operators (F.S., E.G.) following a standardized protocol (Appendix B). Skull segmentation was obtained by manually adjusting the Hounsfield Unit threshold in 3D Slicer until optimal cortical bone delineation was achieved (Fedorov et al., 2012), after which the intracranial cavity was extracted using the WrapSolidify extension (3D Slicer Community, 2020). Incomplete filled foramina, fossae, and sutural gaps were corrected manually to ensure a closed and anatomically coherent endocranial surface. The segmentation was truncated inferiorly at the foramen magnum. ICV was calculated directly from the number of voxels of the segmentation. Intra‐ and inter‐observer consistency of the segmentation procedure was confirmed using intraclass correlation coefficients (ICC) (Appendix C).

### 2.4 Endocast annotation and geometric morphometrics

To comprehensively capture endocranial morphology, each endocast surface was annotated using a combination of fixed anatomical landmarks (ALM), curve semi‐landmarks (CLM), and surface semi‐landmarks (SLM). In total, the endocasts were annotated by 31 ALM, 307 CLM distributed along 34 anatomical curves, and 880 SLM distributed over six endocranial regions (frontal Left and Right, Parietal L, R, Posterior fossa L, R). ALM and CLM were placed manually in 3D Slicer (Appendix D). SLM were subsequently projected and slid in R (R Core Team, 2023) to minimize bending energy relative to the sample mean. This sliding procedure reduced artifactual variation introduced by arbitrary point placement while preserving biologically meaningful shape information (Bardua et al., 2019). Consistency of landmark placement was confirmed using intra‐ and inter‐observer analyses based on ANOVA and MANOVA (Appendix C).

### 2.5 Quantification of suture patency

Calvaria suture patency was quantified for the two coronal‐, the metopic‐, the sagittal‐, and the two lambdoid sutures using a continuous metric rather than a binary open/closed classification. For each suture, closure was measured as one minus the fraction of visible CLM after projection of the skull onto the endocranial surface. Values ranged from 0 (fully patent) to 1 (fully fused). This quantitative approach acknowledged that suture closure, here defined as bone edges gradually approaching each other, was a gradual and spatially heterogeneous process (Appendix D). Suture closure assessment was performed independently by two operators (F.S., E.G.).

### 2.6 Shape alignment, size, and allometry

All landmark configurations were aligned using generalized Procrustes superimposition, removing differences in position, orientation, and scale (Klingenberg, 2013; Klingenberg, 2016; Monteiro, 1999). Shape analyses were conducted on Procrustes coordinates, while size was quantified separately using both centroid size (CS) and ICV. The relationship between CS and ICV was assessed using linear regression to verify that ICV provided a robust proxy for overall endocranial size (*R* ^2^ = 0.960, *F*(1,107) = 2532, *p* < 0.001). To quantify differences in allometry between groups and to measure the proportion of the shape variance explained by size, a multivariate regression analysis was applied on the shape variables using permutation‐based Procrustes ANOVA (*procD.lm* function, *geomorph* package) (Adams & Otárola‐Castillo, 2023). These analyses estimated the proportion of shape variance explained by size and tested the differences in allometric trajectories between groups.

### 2.7 Shape variation and covariation analyses

Principal component analysis (PCA) was applied to the Procrustes coordinates to summarize major axes of endocranial shape variation (Adams et al., 2004; Klingenberg, 2016). Shape changes along principal components were visualized using thin plate spline deformation of the endocast that is closest to the mean shape. Two blocks partial least‐squares (2B‐PLS) analyses were used to quantify covariation between (I) shape and age, (II) shape and size, and (III) shape and suture patency (*pls2b* function, *Morpho* package) (Lê et al., 2008), in both populations separately. These analyses allowed assessment of whether variation in sutural closure patterns was associated with coordinated changes in endocranial morphology. Group differences in suture closure trajectories were tested using multivariate analyses of variance (MANOVA) and analyses of covariance (ANCOVA), with log10(age) included as a covariate. As suture closure is known to correlate strongly with age, it is probable that age is a confounder for the effect of suture closure on shape variation. To counteract this effect, a linear regression model on log10(age) was implemented for each suture to predict control closure patterns. Next, the deviation of each Crouzon patient from this trend was computed, resulting in the residual variability irrespective of age, referred to as “suture residuals.” To study the relationship between shape and synostosis pattern, 2B‐PLS was computed between all suture residuals in both groups and Procrustes coordinates. 2B‐PLS was then applied to each suture separately to the Procrustes coordinates in both groups.

### 2.8 Morphological clusters

To evaluate correspondence between endocast shape and qualitative synostosis pattern, as to link our quantitative approach to a clinical perspective, a hierarchical clustering on principal components (HCPC) was performed on shape variables from the Crouzon population (*FactoMineR* package) (Schlager, 2017). Cluster phenotypes were described qualitatively, based on the shape of the paragon (the individual of a cluster that is diverging the most from the other clusters, representing the most extreme case) and the typical (the individual that falls the closest to the mean shape of its cluster) for each cluster. The number of clusters created by the HCPC was chosen when adding one more cluster only added a nonsignificant inertia gain—that is when creating a new cluster did not bring any further relevant shape information (Husson et al., 2010). One senior craniofacial surgeon (RHK) sorted each included Crouzon CT scan into one of the following clinical phenotypic categories: (A) round, (B) moderately brachycephalic, (C) brachy−/turricephalic, (D) scaphocephalic, (E) scaphocephalic with protruding bregma. Suture closure patterns were calculated and visualized for each of three morphological HCPC clusters (1–3) and each of five clinical phenotypic categories (A–E). An unpaired *t*‐test was performed to test whether morphological clusters and clinical categories were associated with degree of closure of each individual closure, with Bonferroni correction for multiple testing. Contingency tables were built to confront clusters based on morphological data or suture closure, and clinical categories. Fisher's exact test was applied to test for association between categorical variables. This test was preferred over chi‐square tests due to small, expected cell counts in several contingency table cells. Similarly, Fisher's exact test was applied to test for association between morphological cluster and FGFR2 protein variant.

All statistical analyses were performed in R (R Core Team, 2023), using the *geomorph* and *Morpho* packages (Adams & Otárola‐Castillo, 2023; Schlager, 2017).

## 3 RESULTS

### 3.1 Size, allometry, and shape

#### 3.1.1 Size

Results of the ANCOVA showed that ICV increased with age in both controls and Crouzon patients (F1,105 = 228.4, *p* < 0.001). The slope did not differ significantly between groups (F1,105 = 1.287, *p* = 0.259), indicating comparable volumetric growth rates (Appendix A).

#### 3.1.2 Allometry

When including both patients and controls, age and volume significantly predicted endocast shape (age: *R* ^2^ = 0.0394, *F*1,107 = 0.961, *p* < 0.001; volume: *R* ^2^ = 0.0300, *F*1,107 = 3.308, *p* < 0.001). Thus, 3.94% of total shape variance was explained by age and 3.00% by volume. MANCOVA of Procrustes coordinates revealed an interaction between group and volume (*R* ^2^ = 0.018, *F*1,105 = 2.112, *p* = 0.014), indicating different allometric patterns between control individuals and Crouzon patients. In contrast, the interaction between group and age was not significant (*R* ^2^ = 0.116, *F*1,105 = 1.385, *p* = 0.143), suggesting parallel age‐related shape changes across groups. Consistently, 2B‐PLS analyses between shape and log10(age) yielded significant correlations in both controls (PLS1 axis = 0.843, *p* = 0.001) and Crouzon patients (PLS1 axis = 0.644, *p* = 0.001).

#### 3.1.3 Shape variation

**FIGURE 2:**
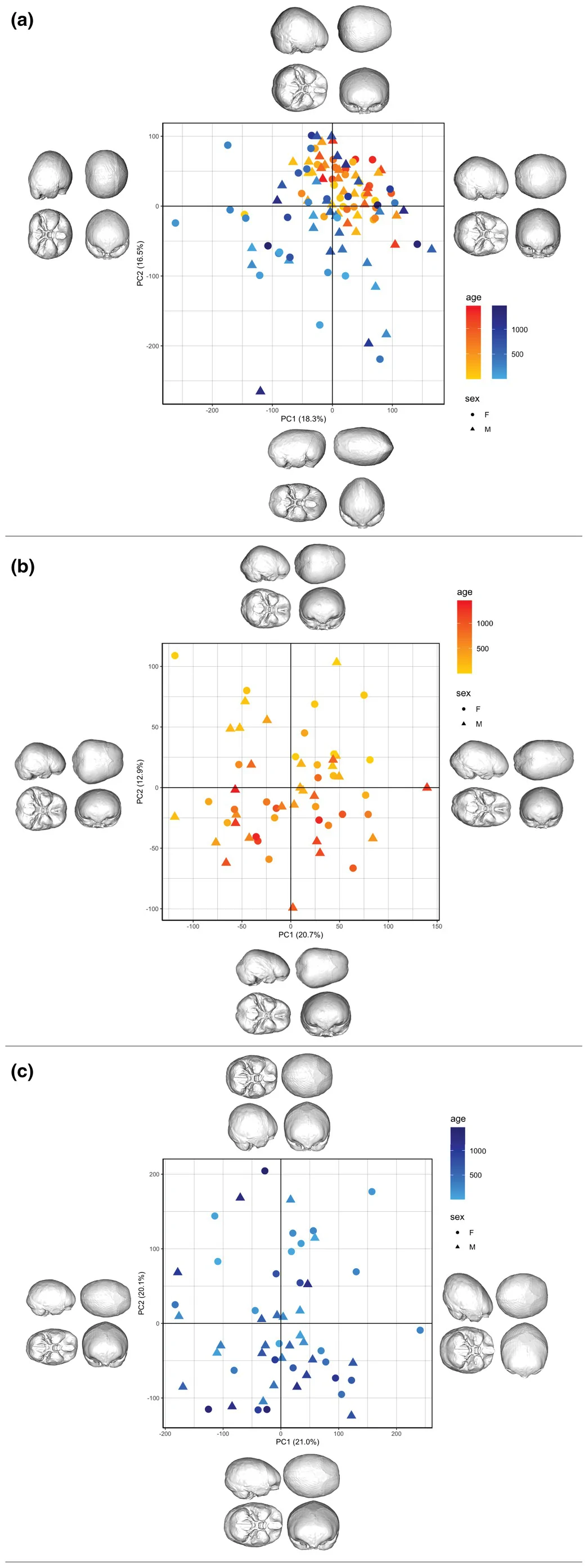
The first two principal components (PCs) of endocranial shape variation in all included specimens and for separate groups. (a) The entire dataset including all control (orange) and Crouzon (blue) endocast samples, (b) control samples only, and (c) Crouzon samples only. Shading corresponds to age at scan. Circles: Boys; triangles: Girls. Theoretical shapes corresponding to the minimal and maximal PC scores are depicted at the extremities of the axes.

The first two principal components of endocranial shape variation for all individuals accounted for 34.8% of total variance (Figure 2a). Ninety‐five percent of the total variance was explained by the first 37 PCs. PC1 (18.3%) primarily reflected variation in overall endocast elongation, ranging from short shapes at negative values to anteroposteriorly elongated shapes at positive values. PC2 (16.5%) captured a more oblique deformation, with an upturned posterior endocast at positive values and an upturned anterior portion with increased anteroposterior length at negative values. Along PC2, a relative decrease in the size of the foramen magnum and cribriform plate was also observed from positive to negative values. Overall, control individuals tended to occupy more positive values along both PC1 and PC2, whereas Crouzon patients were more widely distributed and showed a shift toward negative values. The Crouzon group exhibited greater shape variance than the control population.

In the PCA restricted to controls, PC1 (20.7% of variance) reflected modest changes in anteroposterior dimension, while PC2 (12.9% of variance) predominantly affected the anterior endocast, with a pointier anterior segment at negative PC2 values (Figure 2b). Along with PC2, older individuals tended to show more negative scores, whereas younger individuals displayed more positive scores.

In Crouzon patients (Figure 2c), PC1 (21.0% of variance) accounted for variation from scaphocephalic shapes at negative values to oxycephalic shapes at positive values. Positive PC1 values were associated with a flattened posterior side and a pointed superior margin, whereas negative values correspond to rounder posterior contours. PC2 (20.1% of variance) reflected variation in the relative volumes of the anterior and posterior cranial fossae, with positive values indicating reduced volumes and a shorter anteroposterior diameter, and negative values indicating elongation. Shape deformations along these axes were more pronounced than those observed in controls, indicating higher residual shape variance after accounting for age. Accordingly, endocast shape in the Crouzon population appeared less tightly constrained by allometric effects.

#### 3.1.4 Developmental trajectories

**FIGURE 3:**
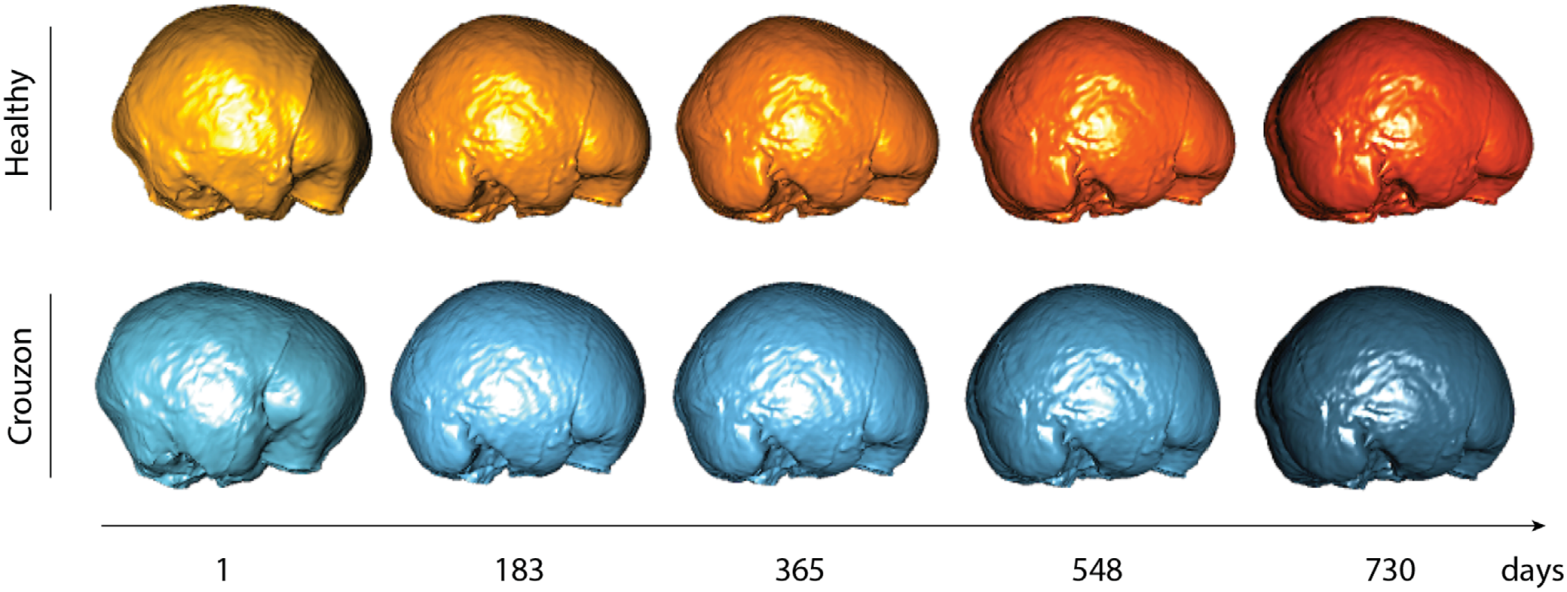
Predicted shapes along the PLS axis through time. Projections shown in lateral view at 6‐month intervals (x‐axis: Age in days) from left to right, for the healthy (top line) and Crouzon (bottom line) groups.

**FIGURE 4:**
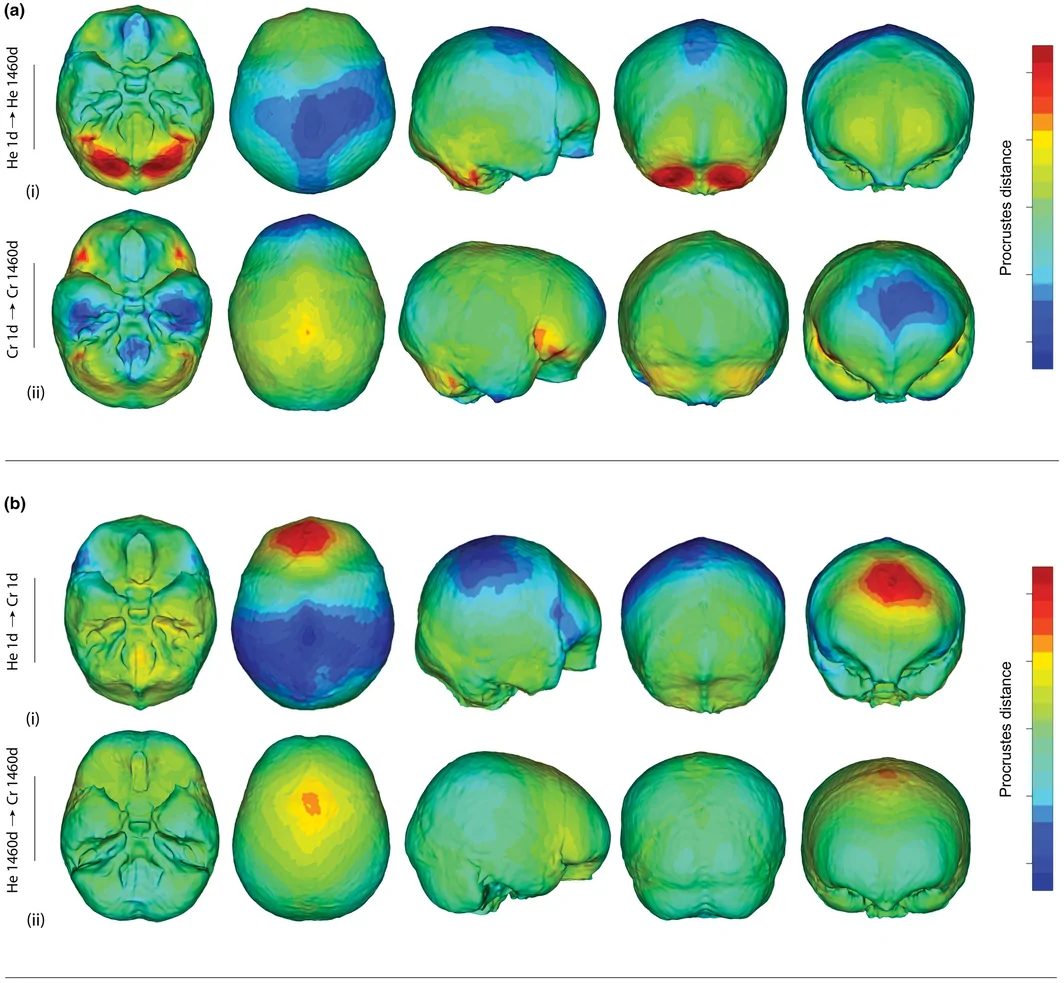
Heatmaps representing areas of variability in endocast shape in inferior, superior, lateral, and posterior views, as warped from one reference endocast. Above (a) through age, as a function of distance from endocast configurations from 0 to 4 years of age for the (i) control and (ii) Crouzon populations, and below (b) between groups, as a function of distance between the endocast configurations plotted on a control endocast at (i) 0 years and (ii) 4 years of age. Cr = Crouzon, He = Healthy. Positive distances (red) describe protrusion with respect to the control mean shape in dimensionless Procrustes distance, whereas negative distances (blue) describe retrusion.

In both controls and Crouzon syndrome patients, the largest shape differences occur during the earliest interval (0–6 months) (Figure 3). In controls, the most prominent changes were localized to the posterior cranial fossa, whereas Crouzon patients showed a progressive general shortening of the entire endocast through time (Figure 4).

### 3.2 Synostosis pattern

**FIGURE 5:**
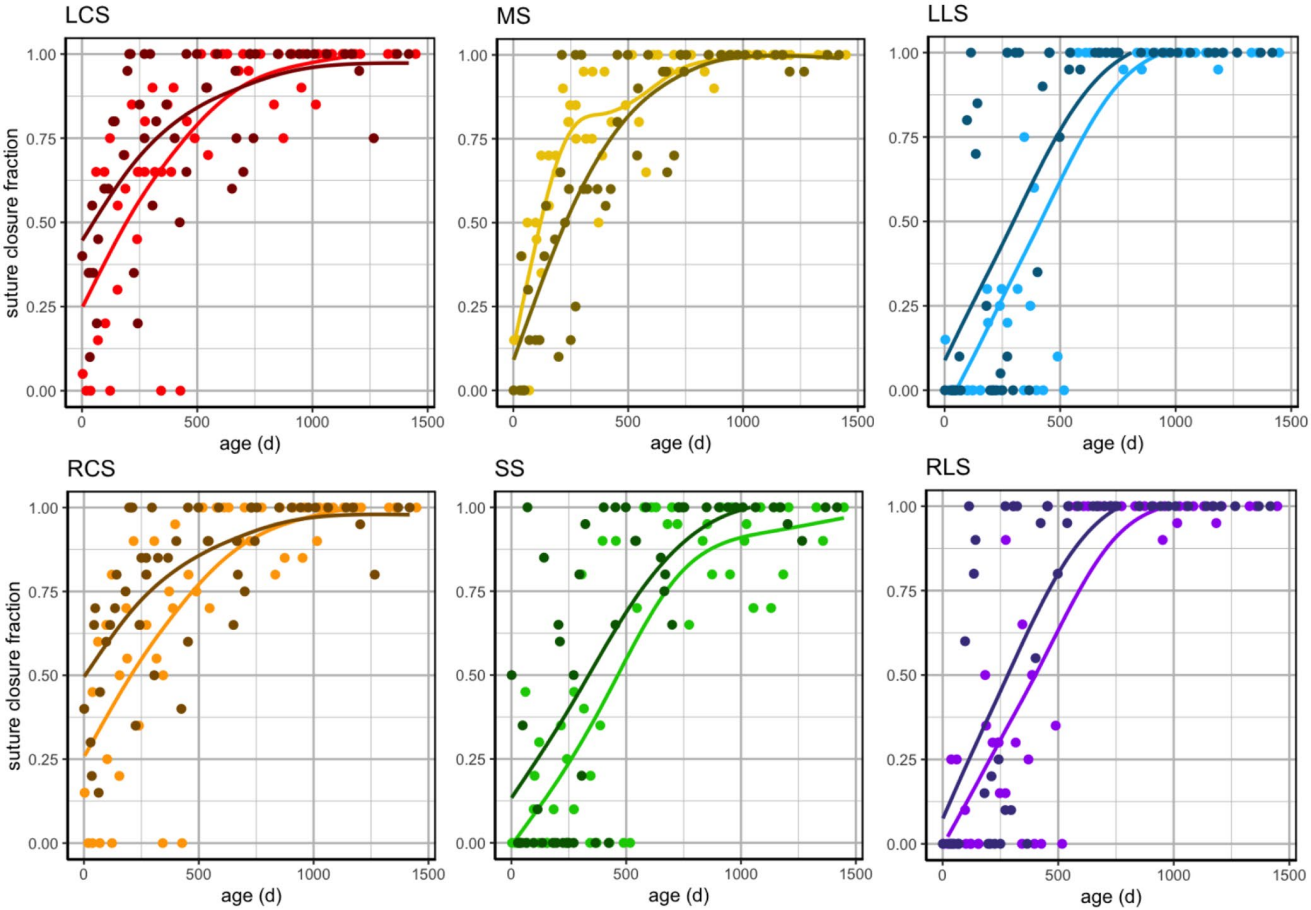
Suture patency fractions as a function of age in the control (light colors) and Crouzon (dark colors) populations. Fractions equate fully open (0) up to fused sutures (1). Trend lines are based on a generalized additive model (GAM). LCS = left coronal, RCS = right coronal, MS = metopic, SS = sagittal, LLS = left lambdoid, RLS = right lambdoid suture.

MANCOVA revealed significant effects of group (Pillai = 0.216, *F* = 4.603, *p* < 0.001) and age (Pillai = 0.757, *F* = 51.821, *p* < 0.001) on overall suture closure patterns (Figure 5, Appendix E). However, the interaction between group and age was not significant (Pillai = 1.07, *F* = 2.001, *p* = 0.072), indicating broadly similar age‐related fusion trajectories in both groups.

When sutures were examined individually, ANCOVA revealed significantly earlier closure of the coronal sutures in Crouzon patients only (left coronal suture: *R* ^2^ = 0.249, *F*1,105 = 6.176, *p* = 0.015; right coronal suture: *R* ^2^ = 0.278, *F*1,105 = 6.905, *p* = 0.010). No significant group‐by‐age interactions were detected for the remaining sutures.

### 3.3 Correspondence between endocast shape and suture patency

#### 3.3.1 Morphological clusters

**FIGURE 6:**
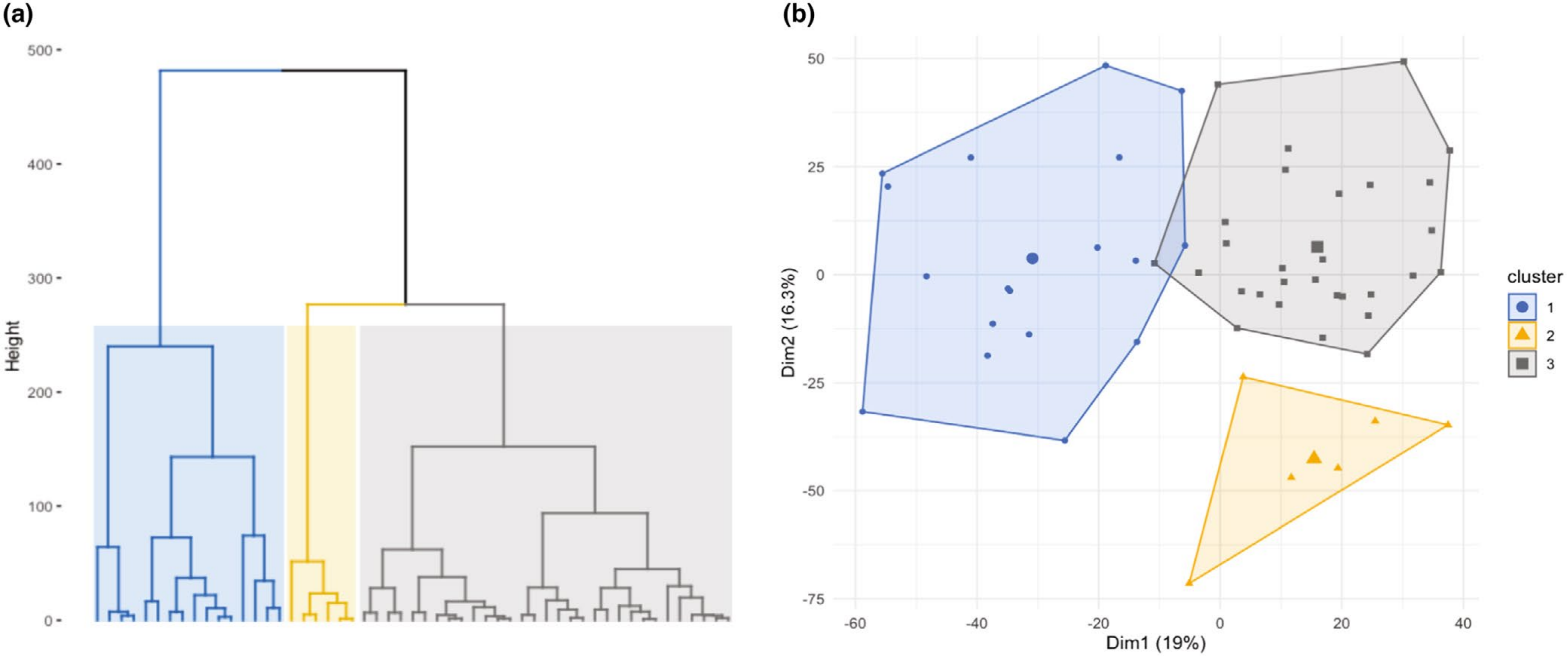
Hierarchical clustering on principal components (HCPC) based on scaled generalized Procrustes coordinates (GPA) of Crouzon individuals showing (a) the clustering dendrogram and (b) annotation in the morphospace of PC1 and PC2. Cluster 1 = blue circles), 2 = yellow triangles, 3 = gray squares. Color fills of surface areas per cluster are for visualization purposes only.

**FIGURE 7:**
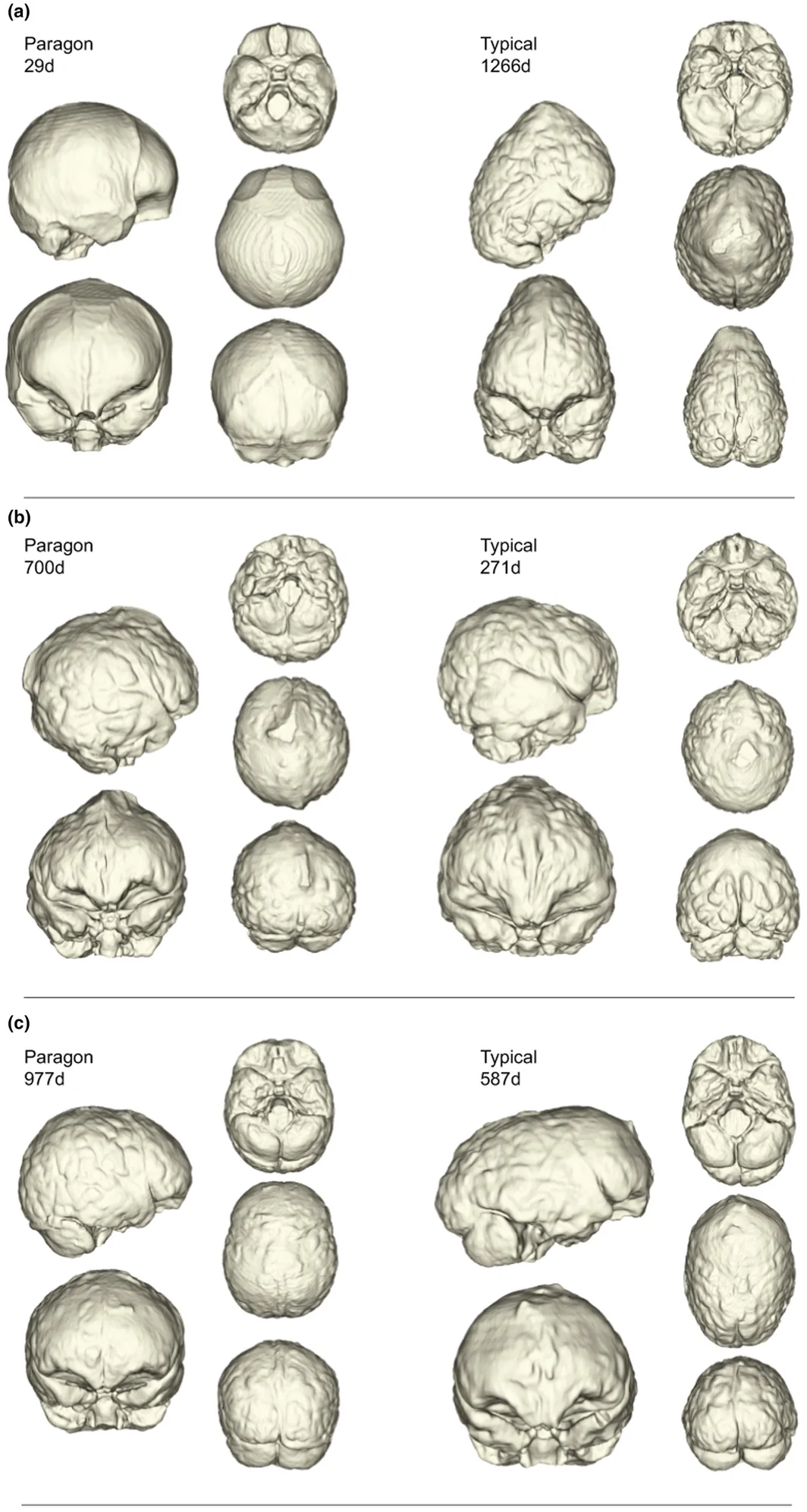
Representative specimens in lateral, frontal, inferior, superior, and posterior views illustrating each morphological cluster (a = 1, b = 2, and c = 3). Paragons (the most extreme case) on the left and typicals (the shape closest to the mean) on the right. Age (days) at CT scan is given for each endocast.

Three clusters were identified and were primarily separated along the first two principal components, accounting for 19.0% and 16.3% of total variance in the Crouzon shape dataset, respectively (Figure 6). Cluster 1 (*n* = 18) was characterized by brachycephalic to turricephalic endocast shapes, with relatively short anteroposterior dimensions and an increased supero‐inferior dimension. Cluster 2 (*n* = 29) displayed a relatively brachycephalic morphology with a visible metopic ridge in both typical and paragon. Cluster 3 (*n* = 6) exhibited elongated anteroposterior dimensions consistent with scaphocephalic shapes (Figure 7).

#### 3.3.2 Influence of suture closure on shape

In control individuals, variation in overall suture patency relative to age expectations did not have a significant effect on endocast shape (rPLS = 0.59, *p* = 0.277). However, when individual sutures were examined separately, all sutures showed significant covariation with shape in 2B‐PLS analyses.

For the coronal sutures, endocasts associated with sutures that were less fused than expected for age showed shortening of the anteroposterior dimension (left and right coronal sutures: rPLS = 0.54, *p* = 0.001 for both). For the metopic suture, endocasts associated with greater‐than‐expected patency showed a flattened forehead in superior view, while lateral views preserved a normal frontal convexity; conversely, more advanced‐than‐expected closure was associated with a downward‐turned anterior endocast segment (rPLS = 0.52, *p* = 0.001). For the sagittal suture, differences in relative patency were not associated with marked anteroposterior elongation of the endocast (rPLS = 0.58, *p* = 0.001). For the lambdoid sutures, endocasts associated with greater‐than‐expected patency showed a narrowing of the anterior segment relative to the middle segment, whereas more advanced closure corresponded to a more balanced proportion between these regions (left lambdoid: rPLS = 0.51; right lambdoid: rPLS = 0.57; both *p* = 0.001).

In the Crouzon population, variation in suture patency relative to age expectations had more pronounced effects on endocast shape. When all sutures were considered jointly, individuals whose sutures were, on average, less fused than expected for age exhibited a shortened endocast with an upturned posterior segment and increased superoinferior dimension (rPLS = 0.69, *p* = 0.038). In contrast, individuals whose overall suture closure was more advanced than expected relative to control reference trajectories exhibited elongated, scaphocephalic endocast shapes.

Analyses of individual sutures revealed significant shape associations for all sutures. For the coronal sutures, endocasts associated with greater‐than‐expected patency showed overall anteroposterior shortening, despite relative elongation of the anterior segment (left: rPLS = 0.66; right: rPLS = 0.69; both *p* = 0.001). For the metopic suture, more advanced‐than‐expected closure was associated with a pointier anterior segment in lateral view, without marked changes in superior view (rPLS = 0.63, *p* = 0.001). In contrast to control individuals, variation in sagittal suture patency in Crouzon patients had strong effects on anteroposterior endocast length: greater‐than‐expected patency was associated with pronounced elongation and scaphocephalic morphology, as well as bulging of the bregma (rPLS = 0.59, *p* = 0.001). For the lambdoid sutures, endocasts associated with greater‐than‐expected patency showed rounder posterior contours in lateral view (left and right lambdoid sutures: rPLS = 0.44, *p* = 0.001).

#### 3.3.3 Clusters, clinical categories, and suture closure

**FIGURE 8:**
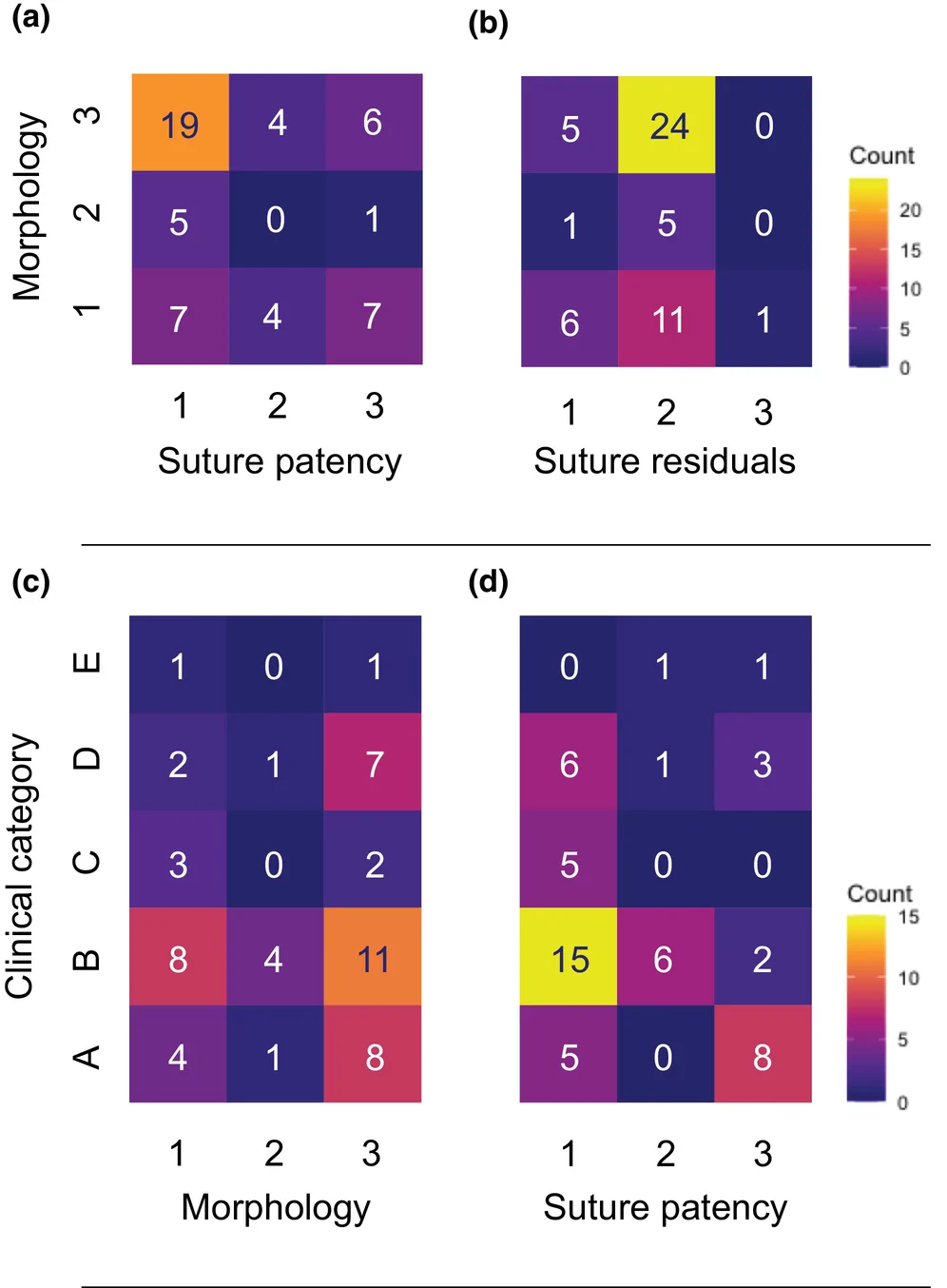
Contingency tables of all Crouzon individuals between assigned morphological clusters and clinical categories. Above between morphological clusters against clusters on (a) suture patency and (b) residual suture patency. Below between clinical categories and clusters on (c) morphology and (d) suture patency. Clinical categories include (A) round, (B) moderately brachycephalic, (C) brachy−/turricephalic, (D) scaphocephalic, (E) scaphocephalic with protruding bregma. The brightness of each cell corresponds to the frequency of individuals in the respective combination of categories/clusters.

**FIGURE 9:**
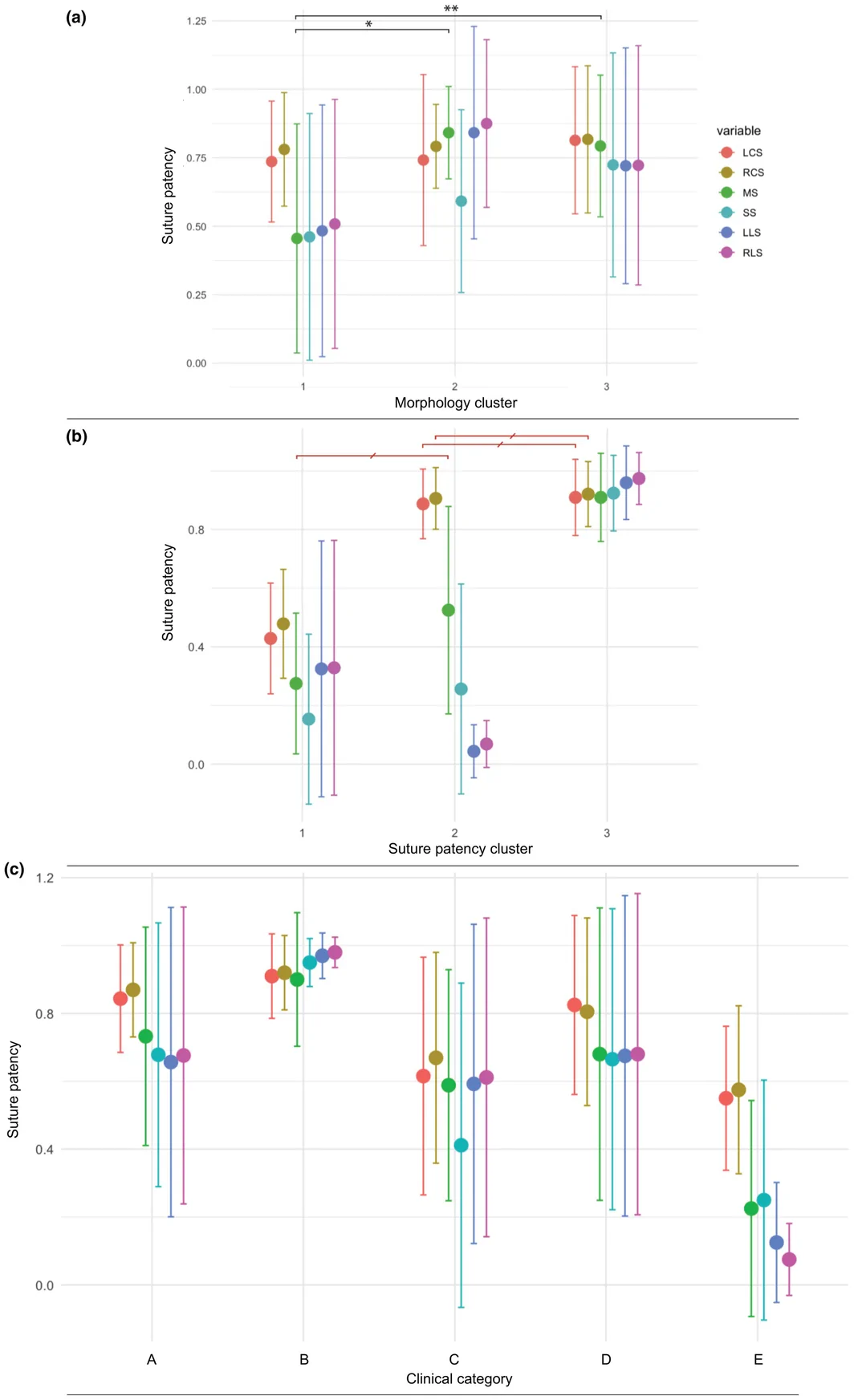
Suture patency fractions (mean ± SD) per suture for each cluster or category. (a) Morphological clusters (*n* = 18,6,29, respectively), (b) clusters based on suture patency (*n* = 14,8,31, resp.), and (c) clinical categories (*n* = 23,5,13,10,2, resp.). Clinical categories include (A) round, (B) moderately brachycephalic, (C) brachy−/turricephalic, (D) scaphocephalic and (E) scaphocephalic with protruding bregma. Suture patency fractions equate fully open (0) up to fused sutures (1). Significance threshold of *p < 0.05 and **p < 0.01 after Bonferroni correction for multiple testing. Note that for plot (b) only nonsignificant relations are indicated above; all other pairs differed significantly. In (c) no pairs reached significance after correction.

**FIGURE 10:**
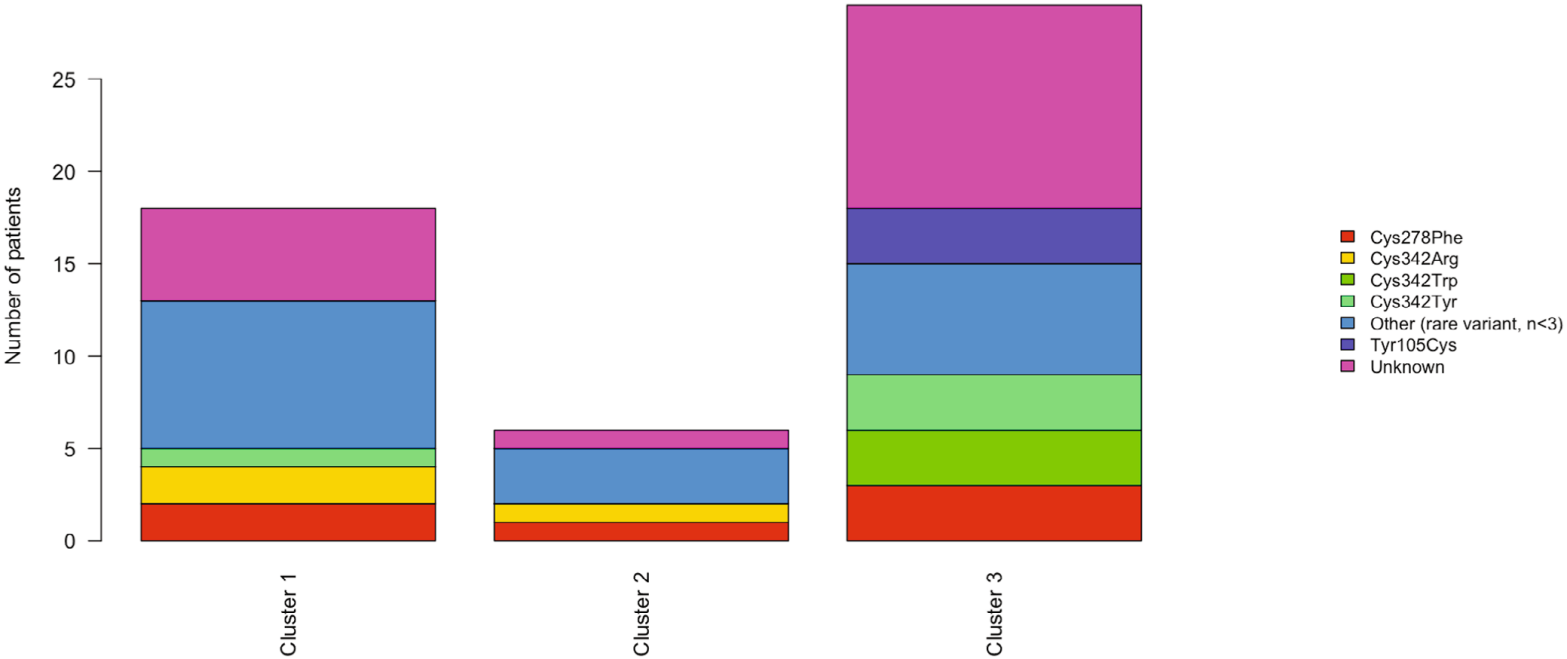
Protein variants of Crouzon patients, shown per morphological cluster. Most common protein variants are shown, along with unknown variants and other rare variants (*n* < 3 in our Crouzon population).

Fisher's exact tests indicated no significant association between intracranial morphology clusters and suture closure patterns (*p* = 0.29), nor between clusters and clinical assessment categories (*p* = 0.87) (Figure 8). Apart from the metopic suture, which was more fused in clusters 2 and 3 compared with cluster 1 (*p* = 0.037 and *p* = 0.002, respectively), no specific suture pattern supported the morphological clustering after Bonferroni correction. No significant differences in suture closure scores were observed between clinical categories proposed by the experts (Table 1, Figure 9). Furthermore, Fisher's exact test showed no significant association between FGFR2‐protein‐variant and intracranial morphology clusters (*p* = 0.138) (Figure 10).

**TABLE 1:** Mean and standard deviations of suture closure scores^a^ for each morphological cluster and clinical category.

	LCS	RCS	MS	SS	LLS	RLS
Morphological cluster						
Cluster 1	0.74 ± 0.22	0.78 ± 0.21	0.46 ± 0.42	0.46 ± 0.45	0.48 ± 0.46	0.51 ± 0.45
Cluster 2	0.74 ± 0.31	0.79 ± 0.15	0.84 ± 0.17	0.59 ± 0.33	0.84 ± 0.39	0.88 ± 0.31
Cluster 3	0.81 ± 0.27	0.82 ± 0.27	0.79 ± 0.26	0.72 ± 0.41	0.72 ± 0.43	0.72 ± 0.44
Suture cluster						
Cluster 1	0.43 ± 0.19	0.48 ± 0.19	0.28 ± 0.24	0.15 ± 0.29	0.33 ± 4,36	0.33 ± 4,34
Cluster 2	0.89 ± 0.12	0.91 ± 0.11	0.53 ± 0.35	0.26 ± 0.36	0.04 ± 0.90	0.07 ± 0.80
Cluster 3	0.91 ± 0.13	0.92 ± 0.11	0.91 ± 0.15	0.92 ± 0.13	0.96 ± 1,25	0.97 ± 0.88
Clinical category						
A. Round	0.84 ± 0.16	0.87 ± 0.14	0.73 ± 0.32	0.68 ± 0.39	0.66 ± 0.46	0.68 ± 0.44
B. Moderate brachycephaly	0.91 ± 0.12	0.92 ± 0.11	0.90 ± 0.20	0.95 ± 0.71	0.97 ± 0.67	0.98 ± 0.45
C. Brachy−/turricephaly	0.62 ± 0.35	0.67 ± 0.31	0.59 ± 0.34	0.41 ± 0.48	0.59 ± 0.47	0.61 ± 0.47
D. Scaphocephaly	0.83 ± 0.26	0.81 ± 0.28	0.68 ± 0.43	0.67 ± 0.44	0.68 ± 0.47	0.68 ± 0.47
E. Scaphocephaly with protruding bregma	0.55 ± 0.21	0.58 ± 0.25	0.23 ± 0.32	0.25 ± 0.35	0.13 ± 0.18	0.75 ± 0.11

^a^ Suture patency fractions equate fully open (0) up to fused sutures (1).

## 4 DISCUSSION

### 4.1 Results interpretation

In this study, the relationship between endocast shape variability and quantitative, continuous suture patency expressed as deviations from control age‐specific expectations (suture residuals) was investigated in Crouzon syndrome where classical models often fail to predict the morphological phenotype (Twigg & Wilkie, 2015b; Flaherty et al., 2016; White et al., 2021). We report that ICV increased strongly with age in both groups with comparable slopes, confirming that intracranial expansion can be preserved in syndromic craniosynostosis during early childhood (Breakey et al., 2018; Fok et al., 1992; Langvatn et al., 2019; Tovetjärn et al., 2014). This supported the now common view that neurological risk cannot be reduced to ICV alone and may instead reflect regional deformation and altered brain–skull interactions (Barbeito‐Andrés et al., 2020; Geoffroy et al., 2022; Richtsmeier & Flaherty, 2013). These findings may have implications for clinical decision‐making, suggesting that volumetric measures alone are insufficient to characterize intracranial risk and that integrating quantitative shape analysis may refine phenotypic stratification in syndromic craniosynostosis.

Furthermore, age and volume explained only a small fraction of shape variance, nevertheless indicating that age‐related shape change was parallel across groups, whereas size–shape allometry differed. This suggested that Crouzon syndrome affected how shape responded to size increase (i.e., altered coupling), rather than grossly changing the temporal ordering of developmental shape changes (Klingenberg, 2016; Richtsmeier & Flaherty, 2013). Crouzon syndrome does not necessarily “shift the clock” of endocranial shape change: it appears to modify the coupling between growth and form. Given the known involvement of the cranial base, dura, and multisuture compensation in syndromic craniosynostosis, one plausible hypothesis is that subtle allometric differences reflect altered distribution of growth‐related strain and compensatory remodeling across vault sutures and cranial base synchondroses, rather than a primary change in the rate of brain expansion itself (Flaherty et al., 2016; Geoffroy et al., 2022; Richtsmeier & Flaherty, 2013). The developing venous system may also contribute to these patterns. Cranial venous architecture is established early and is frequently altered in syndromic craniosynostosis, potentially influencing intracranial pressure dynamics and regional strain fields before or alongside sutural fusion. Subtle allometric differences may therefore reflect the combined effects of sutural mechanics, cranial base growth, and vascular remodeling within an integrated growth system.

In addition, suture closure showed a strong age effect in both groups, but only the coronal sutures exhibited significantly earlier age‐related closure in Crouzon syndrome. This aligned with the Crouzon syndrome phenotype (Johnson & Wilkie, 2011; Renier et al., 2000) and with the consensus that syndromic craniosynostosis frequently involves progressive, multisite fusions with syndrome‐specific suture closure patterns (Flaherty et al., 2016; Hoshino et al., 2022; Pandya et al., 2019; Wang et al., 2021).

Finally, suture residuals covariation with endocast shape was evident in Crouzon syndrome. Suture residuals were associated with major, coherent endocranial shape differences, including anteroposterior shortening/elongation and cranial fossa rebalancing. This result could be expected in a system‐level growth disorder (brain–skull–suture–base) rather than a local–fused‐suture‐based–model (Flaherty et al., 2016; Richtsmeier & Flaherty, 2013; Richtsmeier et al., 2006; Twigg & Wilkie, 2015b; White et al., 2021). Of note, these analyses demonstrate statistical covariation, and while they are consistent with a mechanistic view of coupled growth, they do not prove causality. Cranial fossa reconfiguration may be mechanically linked to premature fusion of cranial base synchondroses, which has been shown to drive progressive craniofacial dysmorphology in syndromic craniosynostosis (Hoshino et al., 2022). Although synchondrosis status was not directly assessed in the present cohort, this association is consistent with the broader view that fossa and vault shape changes in Crouzon syndrome cannot be attributed to vault sutures alone.

### 4.2 Residual suture patency in Crouzon syndrome

Because suture closure was strongly age‐dependent, patency alone was a weak descriptor of underlying mechanisms. Analyzing residuals made the biological interpretation more direct: a negative residual in a Crouzon patient meant that the suture was more fused than expected for a control child of the same age, whereas a positive residual meant that the suture was more patent than expected.

In syndromic craniosynostosis, a suture patent on a CT scan does not necessarily imply that it is functional. Several studies emphasize that sutures are active mechanosensitive organs integrating osteogenic fronts, dural signaling, and local strain fields, with patency governed by interacting molecular and mechanical processes (Greenwald et al., 2000; Khonsari et al., 2013; Menon et al., 2021; Pandya et al., 2019; Wu & Gu, 2019). A suture may remain partially visible yet be mechanically restrictive if local ossification, interdigitation, dural osteogenic signaling, or altered strain environments effectively reduce growth potential. Treating patency as continuous and age‐adjusted is therefore not merely a statistical convenience: it relates to how much a given suture deviates from normal closure dynamics at a given developmental stage, irrespective of age.

Residual patency may thus be interpreted as an indicator of the growth system itself. It is not only a proxy for how fused a suture looks, but also a marker of how the coupled brain–skull–dura system is distributing and resolving growth‐related strain during development (Cross et al., 2021; Flaherty et al., 2016; Richtsmeier & Flaherty, 2013). Under this interpretation, associations between residuals and shape are not related to effects of isolated sutures, but they can be interpreted as signs of the compensatory regime of the system.

### 4.3 Preserved volume, altered shape

These findings reinforce a point increasingly recognized clinically: syndromic craniosynostosis can show relatively preserved ICV while still carrying substantial risk for intracranial hypertension and cognitive developmental impacts (Fok et al., 1992; Langvatn et al., 2019; Tovetjärn et al., 2014; Wilson et al., 2020). This is consistent with the concept that intracranial pressure and cognitive development may depend on regional constraints, altered venous dynamics such as jugular foramina stenosis, posterior fossa/cervicocranial junction configuration, and cranial base involvement, rather than global volume restriction alone (Richtsmeier & Flaherty, 2013; Geoffroy et al., 2022; Lif et al., *personal communications on unpublished manuscript (with permission)*). Crouzon syndrome has well‐described involvement of the foramen magnum and cranial base growth, which can influence posterior fossa anatomy and potentially interact with endocranial shapes (Coll et al., 2012; Dong et al., 2026; Rijken et al., 2013). Thus, ICV trajectories as in controls may be associated with severe clinical presentations and may indicate that growth occurs via spatial and temporal redistribution rather than through global restriction.

### 4.4 Virchow's law revisited: Predictive values and limits

Virchow's law remains a historically foundational statement: growth restriction perpendicular to a fused suture, with compensatory growth parallel to it, seems to predict shapes such as scaphocephaly (due to sagittal suture synostosis) or brachycephaly (due to bicoronal suture synostosis). The current consensus, however, is that it functions best as a first‐order descriptor, suggesting it only accounts for the main component of shape variation, rather than a predictive model (Flaherty et al., 2016; Hoshino et al., 2022; Liu et al., 2023; Menville et al., 2025; White et al., 2021). This limitation is more pronounced in syndromic disease than in single‐suture craniosynostosis, where growth restriction and compensatory patterns more closely follow the classical perpendicular/parallel prediction (Heuzé et al., 2010; Mercan et al., 2020).

Associations such as sagittal suture involvement relating to endocast elongation in Crouzon syndrome resonate with Virchow's law. Yet, the detailed results demonstrate why this law cannot be predictive. The effect of multisuture fusion acts on a system level: endocast shape changes are not confined to a simple perpendicular/parallel deformation around one suture but involve cranial fossae proportions, posterior vault contouring, and integrated cranial base constraints—features reflecting a coupled growth system (Flaherty et al., 2016; Richtsmeier et al., 2006; Richtsmeier & Flaherty, 2013; White et al., 2021). Patency is also graded, spatially heterogeneous, and time‐dependent. Virchow's framing assumes a discrete fused/patent event, whereas continuous residuals explicitly capture partial and age‐dependent deviations (Didziokas et al., 2025; Pandya et al., 2019; Stanton et al., 2022). Syndromic craniosynostosis also includes cranial base and facial growth impairment, meaning vault sutures are only one part of the origins of the skull shape (Flaherty et al., 2016; Hoshino et al., 2022; Pandya et al., 2019; Wang et al., 2021; White et al., 2021). Taken together, these considerations support the view that Virchow's law is best treated as a directional description in isolated craniosynostosis, that must be embedded in a growth disorder framework if mechanistic explanations are expected, especially in Crouzon syndrome (Flaherty et al., 2016; Richtsmeier et al., 2006; Twigg & Wilkie, 2015b).

### 4.5 Why is the suture–shape mapping nontrivial, even with patency quantification?

Even though patency–shape covariation is strong in Crouzon syndrome, the clustering results show that morphological subgroups do not map to specific closure patterns (and clinical categories do not map either). Other studies suggest that similar closure configurations can coincide with different shapes and that closure effects can be nonlocal (Delassus et al., 2026; Liu et al., 2023).

A mechanistic interpretation consistent with both these data and the literature is that sutural status defines boundary conditions, while realized shape depends on the full compensatory regime involving adjacent sutures, cranial base growth, facial growth, bone remodeling, and brain‐driven strain (Flaherty et al., 2016; Mercan et al., 2020; Richtsmeier et al., 2006; White et al., 2021). Modeling work in isolated sagittal synostosis, for example, demonstrates accelerated growth at unfused sutures and quantifies compensatory growth, suggesting that what matters is not simply whether a suture is fused, but how growth is redistributed across remaining interfaces (Mercan et al., 2020). Syndromic cases likely amplify this complexity through multisite involvement and altered material/biological properties, including potential brain anomalies (Delassus et al., submitted).

Given this, the nontrivial suture to shape mapping may be an expected property of a compensated growth system rather than noise. In such systems, multiple internal configurations (patency patterns, timing, cranial base involvement) can yield similar external outcomes (many‐to‐one mapping), and conversely similar shapes can arise through distinct developmental routes (one‐to‐many mapping). A practical consequence is that prediction may require multimodal state variables—such as patency residuals combined with cranial base metrics, shape allometry, and potentially neuroimaging measures—rather than any single descriptor.

### 4.6 Crouzon syndrome as a test case

Quantitative patency should be informative in a setting combining relative genetic homogeneity with pronounced phenotypic dispersion, frequent multisite involvement, and known limits of classic growth theories (Twigg & Wilkie, 2015b; Flaherty et al., 2016; Wang et al., 2021; White et al., 2021). In that context, the strong covariation between residual patency and endocast shape supports the view that continuous patency is relevant and can reveal mechanisms that discrete scoring may obscure.

### 4.7 Limitations

This study is cross‐sectional. Although the age range is dense in early infancy, cross‐sectional inference cannot account for individual trajectories, closure sequences, within‐individual compensation, and age‐dependent severity. CT‐based patency also has resolution and contrast limitations. Slice thickness and reconstruction methods can influence detectability and may introduce measurement error, particularly in advanced fusion stages or with intricate sutural geometries (Massimi et al., 2019). Radiological patency therefore remains an imperfect indicator for functional patency.

Endocast morphology is an indirect indicator of neuroanatomy. Endocasts integrate cranial boundary conditions and provide a meaningful spatial summary, but they do not directly capture regional brain structure, especially regarding sulcation, which is relevant for expecting correlation with neuropsychological parameters (Richtsmeier & Flaherty, 2013). Finally, syndromic craniosynostosis is a multi‐tissue disorder affecting the face and the skull base; hence, vault sutures alone cannot explain the full craniofacial phenotype, notably knowing the major role of synchondroses in anterioposterior growth (Flaherty et al., 2016; Hoshino et al., 2022; Pandya et al., 2019; Wang et al., 2021; White et al., 2021).

### 4.8 Methodological contribution and future perspectives

Quantifying patency as a continuous metric and converting it to age‐adjusted residuals provides a practical way to incorporate sutures into morphometric investigations about causality (Monteiro, 1999). This framework creates a bridge between descriptive morphometrics and mechanistic models that require quantitative inputs, including finite‐element simulations and growth models (Barbeito‐Andrés et al., 2020; Geoffroy et al., 2022). The semiautomated endocast pipeline further supports scalable endocast extraction and reproducible landmarking, which are prerequisites for larger scale studies and multi‐cohort comparisons (Bardua et al., 2019; Fedorov et al., 2012). Although endocasts are not the brain, they remain an integrative anatomical interface reflecting the spatial outcome of brain expansion within cranial constraints and have a long record of use for ontogenetic and comparative analyses (Bienvenu et al., 2011; Bruner & Ripani, 2008; Neubauer et al., 2009).

Future work would benefit from longitudinal designs combining repeated imaging with analyses on continuous patency to infer causal ordering and quantify compensation over time. Integrating cranial base and facial metrics, including synchondroses, is also likely to strengthen mechanistic explanation and improve prediction in syndromic cases (Coll et al., 2012; Flaherty et al., 2016; Rijken et al., 2013). Biomechanical and growth simulations could then use residual patency as an input parameter rather than assuming binary fused/open constraints (Barbeito‐Andrés et al., 2020; Geoffroy et al., 2022; Liu et al., 2023). In this cohort, exploratory analysis of FGFR2 protein variants relative to morphological clusters revealed no significant association. However, power was limited by a heterogeneous set of variants, so larger cohorts remain needed to elucidate the true nature of the relationship. Complementary to longitudinal human studies, genetically homogeneous mouse models of Crouzon syndrome offer an opportunity to isolate the effect of genotype on suture–shape relationships (Perlyn et al., 2006). Ultimately, combining endocast shape, continuous patency residuals, genetics and brain imaging markers may help identify which aspects of intracranial morphology relate most strongly to functional outcomes in syndromic craniosynostosis (Bilal & Shah, 2025; Wilson et al., 2020).

## 5 CONCLUSIONS

In this study, we combined semiautomated three‐dimensional endocast morphometrics with a quantitative, age‐adjusted assessment of calvarial suture patency to examine early cranial development in Crouzon syndrome. We show that ICV is largely preserved, while endocranial shape and its allometric relationship to growth are altered. Continuous suture patency residuals covary strongly with endocast morphology in Crouzon patients, demonstrating that sutural state contributes to phenotypic variability on a system‐level. Taken together, these findings explain why classical suture‐based models such as Virchow's law fail to predict shape in syndromic craniosynostosis: suture effects are graded, age‐dependent, and embedded within a coupled brain–skull vault–skull base growth system.

## AUTHOR CONTRIBUTIONS

Study design: MT; Data Acquisition: FS, EG, OD; Analyses: FS, EG; Interpretation: All authors; Manuscript draft: FS, EG; Manuscript revision and editing: All authors; Supervision: LVdL, MT. All authors approved the last version of the manuscript.

## CONFLICT OF INTEREST STATEMENT

The authors have no conflicts of interest to declare.

## Supporting information


**Appendix A.** ICV and patient characteristics.
**Figure A1**. Scatterplot of trendlines and 95% CI of intracranial volume (ICV). Crouzon = blue, control = red. Girls = circles; boys = triangulars. Trend lines based on nonparametric generalized additive model (GAM).
**Table A1**. ICV measurements and characteristics per patient.
**Appendix B**. Detailed segmentation protocol.
**Figure B1**. Result after skull segmentation. (a) Sagittal view and (b) 3D rendering. N.B.: Here the jaw, dummy, and cervical spine were removed manually, but this is not necessary for the protocol.
**Figure B2**. White arrows indicate areas where the automatic segmentation of the endocast (ivory) often is not primarily satisfactory, after manual adjustments. (A) Cribriform plate, (B) dorsum sellae, (C) foramen magnum, (D) supraorbital fissures.
**Figure B3**. Final results after segmentation and correction of the endocast in 3D view. (a) Endocast in the intracranial cavity, (b) endocast alone.
**Appendix C**. Intra‐ and inter‐observer reliability.
**Table C1**. Intra‐ (a, b) and inter‐observer, (c) variability of total anatomical (ALM) and curve (CLM) landmark placement on three test subjects.
**Appendix D**. Annotation protocol.
**Table D1**. Anatomical landmarks (ALM).
**Table D2**. Semi‐landmarks of curve (CLM).
**Table D3**. Semi‐landmarks of surface (SLM).
**Figure D1**. Anatomical landmarks (red), semi‐landmarks on curves (blue) and surface semi‐landmarks (green) on the endocast of a healthy control in (a) inferior, (b) superior, (c) lateral, (d) posterior, and (e) anterior view.
**Figure D2**. Landmark placement in cross‐sectional views with skull (grayscale) and endocast segmentation (yellow) including the axial (upper left), coronal (bottom left), and sagittal view (bottom right) as well as on 3D rendering (upper right). Each landmark is located at the crosshairs on cross‐sectional views, and at the white arrow in the 3D rendering.
**Figure D3**. Illustration of suture patency quantification. Patency is defined as the fraction of unobscured (a) curve landmarks in open sutures versus the total amount of landmarks in that specific suture (a + b). Example calculation of suture patency in the left coronal suture (LCS) and a graphic illustration of residual suture patency are shown on the right.
**Appendix E**. Specific grades of suture closure.
**Table E1**. Specific grades of suture closure for each sample based on raw data. Suture closure fractions equate fully open (0) up to fused sutures (1).

## Data Availability

The data that support the findings of this study are available from the corresponding author upon reasonable request.
