# Supplementary material for: When Virchow's law fails: Crouzon syndrome as a test case for quantitative suture closure–endocranial shape relationships
Source: J Anat. 2026 Sep 14:10.1111/joa.70237. Online ahead of print. doi: 10.1111/joa.70237 (PMC13575353; doi:10.1111/joa.70237)
Supplement: Supplementary file 1 [file JOA-9999-0-s001.docx]

8. Supplementary material

All R scripts used for geometric morphometric analyses, suture patency quantification, and statistical modelling are publicly available at [GitHub repository URL, to be inserted upon acceptance].

Appendix A. ICV and patient characteristics

**Figure A1.** Scatterplot of trendlines and 95% CI of intracranial volume (ICV). Crouzon = blue, control = red. Girls = circles; boys = triangulars. Trend lines based on non-parametric Generalized Additive Model (GAM).

**Table A1.** ICV measurements and characteristics per patient

|  | **Sex** | **Age (d)** | **Age (m)** | **ICV (mm3)** | **Reason for CT imaging** |
| --- | --- | --- | --- | --- | --- |
| **Control** |  |  |  |  |  |
| **1** | F | 3 | 0 | 394651 | craniofacial trauma |
| **2** | F | 19 | 0 | 469819 | craniofacial trauma |
| **3** | F | 37 | 1 | 427863 | hypotonia |
| **4** | M | 37 | 1 | 608016 | seizure |
| **5** | F | 61 | 2 | 558347 | craniofacial trauma |
| **6** | F | 69 | 2 | 609734 | hypotonia |
| **7** | F | 97 | 3 | 727947 | craniofacial trauma |
| **8** | M | 101 | 3 | 803282 | craniofacial trauma |
| **9** | M | 121 | 4 | 690425 | CMV infection |
| **10** | F | 122 | 4 | 826159 | craniofacial trauma |
| **11** | M | 154 | 5 | 806180 | seizure |
| **12** | M | 155 | 5 | 787235 | CMV infection |
| **13** | F | 184 | 6 | 611557 | seizure |
| **14** | M | 188 | 6 | 914006 | CMV infection |
| **15** | M | 216 | 7 | 959275 | CMV infection |
| **16** | F | 239 | 7 | 900047 | ischemic accident |
| **17** | M | 242 | 7 | 1038880 | abnormal movements |
| **18** | M | 247 | 8 | 1026820 | craniofacial trauma |
| **19** | M | 272 | 8 | 887845 | Head and neck infection |
| **20** | F | 273 | 8 | 954336 | craniofacial trauma |
| **21** | F | 306 | 10 | 985118 | seizure |
| **22** | M | 316 | 10 | 1055230 | craniofacial trauma |
| **23** | F | 343 | 11 | 831482 | craniofacial trauma |
| **24** | F | 345 | 11 | 895642 | Methylmalonic aciduria |
| **25** | F | 371 | 12 | 703985 | craniofacial trauma |
| **26** | M | 387 | 12 | 1154710 | craniofacial trauma |
| **27** | M | 397 | 13 | 981006 | craniofacial trauma |
| **28** | F | 427 | 14 | 1050710 | Craniofacial infection |
| **29** | M | 455 | 15 | v1072580 | craniofacial trauma |
| **30** | M | 489 | 16 | 1113900 | seizures |
| **31** | F | 517 | 17 | 1008880 | mastoiditis |
| **32** | M | 547 | 17 | 1199450 | vomitting |
| **33** | F | 578 | 19 | 1180220 | craniofacial trauma |
| **34** | F | 610 | 20 | 1078180 | headache |
| **35** | M | 630 | 20 | 1258720 | craniofacial trauma |
| **36** | F | 680 | 22 | 1183060 | polytrauma |
| **37** | M | 699 | 23 | 1219330 | craniofacial trauma |
| **38** | F | 723 | 23 | 1253400 | trauma capitis |
| **39** | F | 774 | 25 | 1140800 | craniofacial trauma |
| **40** | F | 833 | 27 | 1257760 | craniofacial trauma |
| **41** | M | 853 | 28 | 1139660 | craniofacial trauma |
| **42** | M | 874 | 28 | 1403080 | craniofacial trauma |
| **43** | M | 912 | 30 | 1436260 | polytrauma |
| **44** | F | 952 | 31 | 1226120 | frontal laceration |
| **45** | M | 960 | 31 | 1140860 | polytrauma |
| **46** | F | 1015 | 33 | 1508250 | craniofacial trauma |
| **47** | M | 1024 | 33 | 1230750 | craniofacial trauma with vomiting |
| **48** | F | 1054 | 34 | 1110960 | craniofacial trauma with vomiting |
| **49** | M | 1085 | 35 | 1149030 | hemophilia |
| **50** | M | 1131 | 37 | 1430660 | craniofacial trauma |
| **51** | F | 1167 | 38 | 1149110 | craniofacial trauma |
| **52** | M | 1184 | 38 | 1558730 | craniofacial trauma with vomiting |
| **53** | M | 1207 | 39 | 1255820 | polytrauma |
| **54** | F | 1328 | 43 | 1234630 | craniofacial trauma with vomiting |
| **55** | F | 1357 | 44 | 1187870 | craniofacial trauma |
| **56** | M | 1448 | 47 | 1284790 | craniofacial trauma with vomiting |
| **Crouzon** |  |  |  |  | **FGFR2 protein variant** |
| **1** | F | 1 | 0 | 499753 | Asn331lle |
| **2** | F | 29 | 0 | 484085 | - |
| **3** | F | 34 | 1 | 516335 | Tyr105Cys |
| **4** | M | 44 | 1 | 707864 | Cys342Arg |
| **5** | F | 49 | 1 | 581692 | Cys278Phe |
| **6** | M | 64 | 2 | 559842 | Phe276Val |
| **7** | M | 70 | 2 | 665168 | - |
| **8** | F | 97 | 3 | 813474 | Cys342Arg |
| **9** | M | 114 | 3 | 576968 | - |
| **10** | F | 135 | 4 | 538936 | Cys342Ser |
| **11** | F | 142 | 4 | 858619 | Cys342Ser |
| **12** | M | 181 | 5 | 677563 | Cys278Phe |
| **13** | M | 197 | 6 | 958891 | Tyr328Cys |
| **14** | M | 205 | 6 | 875784 | - |
| **15** | F | 211 | 6 | 942286 | Asp273delinsGluAsn |
| **16** | M | 225 | 7 | 549197 | - |
| **17** | F | 242 | 7 | 929738 | Cys278Phe |
| **18** | F | 250 | 8 | 978744 | Cys342Tyr |
| **19** | F | 271 | 8 | 1032200 | - |
| **20** | F | 271 | 8 | 1106370 | Ser347Cys |
| **21** | M | 296 | 9 | 915222 | - |
| **22** | M | 306 | 10 | 1240110 | - |
| **23** | M | 322 | 10 | 780096 | - |
| **24** | F | 366 | 12 | 893656 | - |
| **25** | F | 402 | 13 | 1093820 | Cys278Phe |
| **26** | M | 424 | 13 | 1783350 | Ser354Cys |
| **27** | F | 453 | 14 | 1265250 | Ser354Cys |
| **28** | F | 453 | 14 | 977449 | Gln289Pro |
| **29** | M | 498 | 16 | 1115800 | Tyr105Cys |
| **30** | M | 539 | 17 | 1016970 | Cys278Phe |
| **31** | M | 543 | 17 | 1023490 | Cys342Trp |
| **32** | M | 587 | 19 | 990487 | - |
| **33** | F | 651 | 21 | 1319680 | - |
| **34** | F | 666 | 21 | 1181390 | Cys342Arg |
| **35** | M | 670 | 22 | 1132310 | Cys278Phe |
| **36** | M | 700 | 22 | 1316890 | Phe276Val |
| **37** | M | 728 | 23 | 1240760 | Tyr105Cys |
| **38** | M | 729 | 23 | 1089150 | Cys342Trp |
| **39** | F | 744 | 24 | 1340880 | Splice mutation |
| **40** | M | 753 | 24 | 1330340 | Val359Phe |
| **41** | F | 850 | 27 | 1162400 | Tyr340His |
| **42** | M | 904 | 29 | 1294240 | Ala344Ala |
| **43** | F | 943 | 31 | 1178630 | Tyr381Asn |
| **44** | F | 977 | 32 | 1155750 | Cys342Tyr |
| **45** | M | 1008 | 33 | 1207870 | - |
| **46** | F | 1063 | 34 | 1139020 | - |
| **47** | M | 1142 | 37 | 1152370 | Splice mutation |
| **48** | M | 1172 | 38 | 1217670 | - |
| **49** | M | 1203 | 39 | 1388030 | Cys342Tyr |
| **50** | M | 1266 | 41 | 1587300 | - |
| **51** | F | 1368 | 44 | 1186460 | Cys342Tyr |
| **52** | F | 1418 | 46 | 1358840 | Cys342Trp |
| **53** | F | 1478 | 48 | 1086940 | - |

Appendix B. Detailed segmentation protocol

Software: 3D Slicer Image Computing Platform (version 5.6.1)

Required extension: WrapSolidify (3D Slicer Community, 2020)

1. **Import and align**

Module ‘DICOM database’

- Import DICOM image bundle
- Load relevant CT scan, if possible in OS setting (i.e. convolution kernel = ‘BONE’)
- If orientation is very askew, e.g. midsagittal plane is not roughly vertical in coronal view, reorient the volume using the tool ‘Linear Transform’

2. **Skull segmentation**

Module ‘Volumes’ > Submenu ‘Display’ > Set Window/Level to CT-Bone (1000/400 HU)

Module ‘Segment Editor’

- Select option ’Add segment’
- Select effect ‘Threshold’
- Set threshold range from 200 HU to maximum
- If this threshold does not accurately align with bone contours, either over- or undersegmenting, manually adjust the HU threshold
- Select effect ‘Islands’
- Remove small islands: minimum size 1000 voxels
- Illustration of skull segmentation is shown in Figure B1.

**Figure B1**. Result after skull segmentation. (a) Sagittal view and (b) 3D rendering. N.B.: Here the jaw, dummy and cervical spine were removed manually, but this is not necessary for the protocol

3. **Automatic endocast segmentation**

Module ‘WrapSolidify’

- Select the skull segment in top of window: set only this segment on visible and hide all other segments (if applicable)
- Submenu ‘Wrap Solidify’.
- Region: ‘largest cavity’ and set ‘split cavities’ threshold to 15mm

In case the segmentation does not properly extract the endocast, and visibly protrudes through the spinal canal/cribriform plate/sutures, consider raising the ‘split cavities’ threshold. Moreover, in this case, it may be useful to remove structures from the skull segmentation (such as headrests, spine, mandible, etc.) depending on the extent of protrusion.

- Output: ‘new segment’
- Submenu ‘Advanced’ > Smoothing factor: 0
- Submenu ‘Masking’
- Editable area: ‘outside all visible segments’
- Modify other segments ‘overwrite visible’

4. **Manual adjustments**

Module ‘Segment editor’

- Effect ‘Eraser’ and ‘Paint’ > Sphere brush
- Submenu ‘Masking’
- Editable area: ‘outside all visible segments’
- Modify other segments: ‘overwrite visible’
- Closure of foramen magnum
- In coronal view, use spherical eraser brush and draw lines perpendicular to the line from opisthion to basion in multiple slices
- Verify that there are not too many protrusions and irregularities in 3D view
- Verify that hypoglossal canal is included above the cut of foramen magnum
- Visual inspection and manual adjustment
- Verify that the nasal cavity, orbits, and other soft tissue structures are excluded from the segmentation mask
- Verify that no important cavities are excluded:
- Skull base: delineation at foramina, cribriform plate, synchondroses
- Calvarium: object should not protrude through sutures or fontanelles
- Illustration of areas requiring manual adjustments are shown in Figure B2.
- Final results of skull and endocast segmentation are shown in Figure B3.

**Figure B2**. White arrows indicate areas where the automatic segmentation of the endocast (ivory) often is not primarily satisfactory, after manual adjustments. (A) Cribriform plate, (B) Dorsum Sellae, (C) Foramen Magnum, (D) Supraorbital fissures.

**Figure B3**. Final results after segmentation and correction of the endocast in 3D view. (a) Endocast in the intracranial cavity, (b) endocast alone.

5. **Measure, save and export**

Module ‘Quantification’

- For volumetry and surface measurements of created segments

Save:

- Click ‘create Medical Reality Bundle’ (.mrb)
- Export segment to files
- Visible segments only
- File format: OBJ
- Merge into single file
- Coordinate system: LPS
- Apply linear transforms

Appendix C. Intra- and interobserver reliability

Methods

Segmentation was performed by two authors (F.S., E.G.). Repeatability tests were performed on ten endocasts by both authors. The intra-class correlation coefficient (ICC(2,3)) with 95%CI was calculated based on a mean-rating (k=3), absolute agreement, and two-way mixed-effects model [(31,32)](https://www.zotero.org/google-docs/?CC3k1C). To assess the correspondence of the digitization process between the operators, an inter-observer reliability test was also performed using the ICC on the same subjects. Annotation was performed by two authors (F.S., E.G) and the intra-observer and inter-observer reliability for the placement of landmarks was calculated using ANOVA and MANOVA.

Results

The ICC was high for the segmentation process for intra-observer reliability (F.S. 1.000, 95% CI 0.996-1.000, E.G. 1.000, 95% CI 0.997-1.000) and inter-observer variability (1.000, 95% CI 0.999-1.000). ANOVA showed that intra-observer variability of landmark placement for ALM and CLM together was reliable (F.S. R2=0.964, F(2,6)=79.635, p=0.003, E.G. R2=0.958, F(2,6)=68.821, p=0.001). Additionally, inter-observer variability, tested using MANOVA was reliable, (R2=0.788, F(2,15)=27.798, p=0.001). When looking at PC 1 and PC 2, which explain respectively 61.7% and 19.5% of the variation in the dataset, there was a clear grouping of repetitions and distinction between individuals. Thus, all ALM and CLM were included in further analysis.

a	b	c

**Table C1.** Intra- (a, b) and inter-observer (c) variability of total anatomical (ALM) and curve (CLM) landmark placement on three test subjects.

Appendix D. Annotation protocol

Requirements for inclusion of ALM were as follows: 1) the landmark corresponds to an anatomical reference point on the intracranial surface, 2) the landmark is validated in literature, and 3) the landmark could be reproduced in all subjects. A set of 31 ALM (Table D1, Figure D1) was selected from literature and placed on each endocast [(34–37)](https://www.zotero.org/google-docs/?UISHVy). For individuals with open calvarial sutures, these were extrapolated to define the center of the endolambda or endobregma. 14 Curves, or identifiable ridges or structures between ALM, were defined and annotated on each endocast by CLM. This amounted to 307 CLM in total (Table D2, Figure D1). The skull base curve consisting of 19 ALM served as boundary for the subsequent SLM patching procedure, after which the curve was excluded from further statistical analyses. In order to capture the morphological variance in topologically complex areas of the calvarium and cranial base, SLM were placed. Surface patches were annotated on an hemispheric template mesh, bordered by the predefined CLM (Table D3, Figure D1). The SLM on the hemispheric template were projected to the target meshes by non-rigid registration using a thin-plate spline (TPS) algorithm, guided by all 338 ALM and CLM. Curves were slid along tangent vectors and surface points along tangent planes whilst minimizing the total bending energy (*slider3d* function, *Morpho* package) [(38)](https://www.zotero.org/google-docs/?aY90uT). Curve landmarks were annotated more densely than SLM, as these were found to be more accurate along anatomical structures than the projected SLM. Including the 880 SLM, each endocast was annotated with 1218 landmarks in total (Figure D1).

Every (pair of) landmark(s) is shown in the views used for placement in 3D Slicer (Figure D2).

**Table D1.** Anatomical landmarks (ALM)

| **#** | **Landmark** | **Description** |
| --- | --- | --- |
| 1. | Anterior sphenoid spine | Middle anterior margin of the sphenoethmoidal suture adjacent to the cribriform  plate. |
| 2. | Foramen caecum | Superior margin of the foramen anterior to the cribriform plate of the ethmoid bone  and posterior to the frontal bone (i.e. anterior margin of the frontoethmoidal suture). |
| 3. | Dorsum sellae | Superior and midsagittal point of posterior wall of the sella turcica. |
| 4. | Endosphenobasion | Midsagittal point on inferior margin of the spheno-occipital synchondrosis. |
| 5. | Basion | Anterior margin of the foramen magnum. |
| 6. | Opisthion | Posterior margin of the foramen magnum. |
| 7, 8. | Anterior clinoid process^a^ | Most distant point of bony structure on the superolateral aspect of the sphenoid  sinus, in between the internal carotid artery and the optic nerve. |
| 9, 10. | Optic canal^a^ | Anterior inferior midpoint of optic nerve entrance into the sella turcica. |
| 11, 12. | Superior orbital fissure^a^ | The medial margin of the communication between the cavernous sinus and the apex  of the orbit. |
| 13, 14. | Foramen rotundum^a^ | Midpoint of the opening in the base of the greater wing of the sphenoid bone, found  posterior to the medial end of the superior orbital fissure. |
| 15, 16. | Foramen ovale^a^ | Midpoint of the opening located at the base of the greater wing of the sphenoid,  positioned posterolaterally to the foramen rotundum. |
| 17, 18. | Pyramidal apex^a^ | Superior point of pyramid-shaped anteromedial margin of the petrous part of the  temporal bone. |
| 19, 20. | Internal acoustic meatus^a^ | Middle of the bony passage within the petrous part of the temporal bone, at the  entrance of the vestibulocochlear nerve to the posterior fossa. |
| 21, 22. | Pyramidal base^a^ | Maximum curvature of the petrous curve in the axial plane. |
| 23, 24. | Foramen jugulare^a^ | Most anteromedial margin of the foramen, formed anteriorly by the petrous part of  the temporal bone and posteriorly by the occipital bone. |
| 25, 26. | Hypoglossic canal^a^ | Middle of the canal just above the foramen magnum, through which CN XII passes  to exit the posterior cranial fossa. |
| 27. | Internal occipital protuberance | Junction of the largest sinuses above cerebellum, i.e. confluens of sinus torcular  herophili. |
| 28. | Endolambda | Where the lambdoid sutures meet the sagittal suture. |
| 29. | Endobregma | Where the coronal sutures meet the sagittal suture. In case of an open bregma,  trajectories of the coronal and sagittal sutures are extrapolated. |
| 30, 31. | Sylvian fissure^a^ | Where the posterior margin of the anterior cranial fossa (as extension of the lesser  sphenoid wing) fuses with the lateral wall of the frontal bone. |

*^a^ left- and rightsided ALM*

**Table D2.** Semi-landmarks of curve (CLM)

| # | Curve | # points | Included ALM |
| --- | --- | --- | --- |
| 1. | Metopic suture | 20 | 2, 29 |
| 2. | Midsagittal curve | 30 | 29, 28, 27 |
| 3. | Internal occipital crest | 6 | 27, 6 |
| 4, 5. | Left coronal suture | 20 | 29, 30 |
|  | Right coronal suture | 20 | 29, 31 |
| 6, 7. | Left Transverse Sinus  Right Transverse Sinus | 15  15 | 27, 21  27, 22 |
| 8. | Foramen magnum | 30 | 5, 6, 5 |
| 9. | Skull base | 91 | 25, 23, 19, 17, 15, 13 11, 7, 9, 1, 10, 8, 12, 14, 16, 18, 20, 24, 26 |
| 10, 11. | Left sphenoid curve | 10 | 30, 7 |
|  | Right sphenoid curve | 10 | 31, 8 |
| 12, 13. | Left petrous curve | 10 | 21, 17 |
|  | Right petrous curve | 10 | 22, 18 |
| 14. | Cribriform plate | 20 | 2, 1, 2 |

*^a^ in order of placement*

*^b^ 4 landmarks were placed in between each successive anatomical landmark, along the line of shortest distance on the surface*

**Table D3.** Semi-landmarks of surface (SLM)

| # | Surface patch | # points | Bordering curves | Description |
| --- | --- | --- | --- | --- |
| 1. | Left anterior | 55 | 1, 14, 9, 10, 4 | Frontal bone and anterior cranial fossa |
| 2. | Right anterior | 55 | 1, 14, 9, 11, 5 |  |
| 3. | Left middle | 114 | 2, 4, 10, 9, 12, 6 | Temporal and parietal bones, middle cranial fossa |
| 4. | Right middle | 114 | 2, 5, 11, 9, 13, 7 |  |
| 5. | Left posterior | 26 | 3, 6, 12, 9, 8 | Occipital bone, posterior cranial fossa |
| 6. | Right posterior | 26 | 3, 7, 13, 9, 8 |  |

a	b

c	d	e

**Figure D1.** Anatomical landmarks (red), semilandmarks on curves (blue) and surface semilandmarks (green) on the endocast of a healthy control in (a) inferior, (b) superior, (c) lateral, (d) posterior and (e) anterior view.

| 1. Anterior sphenoid spine (ass)   | 2. Foramen Caecum (fc)   |
| --- | --- |
| 3. Dorsum sellae   | 4. Endosphenobasion (esb)   |
| 5. Basion (bas)   | 6. Opisthion (opi)   |
| 7,8. Anterior clinoid process (l_acp, r_acp)   | 9,10. Optic canal (l_oc, r_oc)   |
| 11, 12. Superior orbital fissure (l_sof, r_sof)   | 13, 14. Foramen rotundum (l_fr, r_fr)   |
| 15, 16. Foramen ovale (l_fo, r_fo)   | 17, 18. Pyramidal apex (l_pa, r_pa)   |
| 19, 20. Internal acoustic meatus (l_iam, r_iam)   | 21, 22. Pyramidal base (l_pb, r_pb)   |
| **23, 24. Foramen jugulare (l_fj, r_fj)**   | 25, 26. Hypoglossic canal (l_hc, r_hc)   |
| 27. Internal occipital protuberance (iop)   | 28. Endolambda (el)   |
| 29. Endobregma (eb)   | 30, 31. Sylvian fissure (l_syf, r_syf)   |

**Figure D2.** Landmark placement in cross-sectional views with skull (grayscale) and endocast segmentation (yellow) including the axial (upper left), coronal (bottom left), and sagittal view (bottom right) as well as on 3D rendering (upper right). Each landmark is located at the cross-hairs on cross-sectional views, and at the white arrow in the 3D rendering.

**Figure D3.** Illustration of suture patency quantification. Patency is defined as the fraction of unobscured (a) curve landmarks in open sutures versus the total amount of landmarks in that specific suture (a+b). Example calculation of suture patency in the left coronal suture (LCS) and a graphic illustration of residual suture patency are shown on the right.

Appendix E. Specific grades of suture closure

**Table E1.** Specific grades of suture closure for each sample based on raw data. Suture closure fractions equate fully open (0) up to fused sutures (1).

|  | Control | |  |  |  |  |  | Crouzon | |  |  |  |  |
| --- | --- | --- | --- | --- | --- | --- | --- | --- | --- | --- | --- | --- | --- |
|  | LCS | RCS | MS | SS | LLS | RLS |  | LCS | RCS | MS | SS | LLS | RLS |
| 1. | 0,95 | 0,85 | 0,85 | 1,00 | 0,85 | 1,00 |  | 0,60 | 0,60 | 1,00 | 0,50 | 1,00 | 1,00 |
| 2. | 1,00 | 1,00 | 0,85 | 1,00 | 1,00 | 1,00 |  | 0,65 | 0,70 | 1,00 | 1,00 | 1,00 | 1,00 |
| 3. | 0,65 | 0,55 | 0,60 | 1,00 | 1,00 | 0,75 |  | 0,90 | 0,80 | 0,60 | 1,00 | 1,00 | 1,00 |
| 4. | 1,00 | 1,00 | 1,00 | 1,00 | 1,00 | 1,00 |  | 0,45 | 0,35 | 1,00 | 1,00 | 1,00 | 1,00 |
| 5. | 0,35 | 0,40 | 0,50 | 0,55 | 1,00 | 0,75 |  | 0,65 | 0,30 | 1,00 | 0,65 | 1,00 | 1,00 |
| 6. | 0,85 | 1,00 | 1,00 | 1,00 | 1,00 | 1,00 |  | 0,80 | 0,85 | 0,70 | 1,00 | 0,90 | 1,00 |
| 7. | 0,35 | 0,35 | 0,50 | 0,90 | 1,00 | 0,90 |  | 0,55 | 0,55 | 0,85 | 0,00 | 1,00 | 1,00 |
| 8. | 0,80 | 0,75 | 0,55 | 0,80 | 1,00 | 1,00 |  | 0,40 | 0,40 | 0,85 | 1,00 | 0,20 | 0,40 |
| 9. | 0,25 | 0,20 | 0,30 | 0,70 | 1,00 | 1,00 |  | 0,40 | 0,35 | 0,85 | 0,90 | 0,00 | 0,00 |
| 10. | 1,00 | 1,00 | 0,65 | 1,00 | 1,00 | 1,00 |  | 0,20 | 0,30 | 0,60 | 1,00 | 0,30 | 0,20 |
| 11. | 0,70 | 0,80 | 0,30 | 1,00 | 1,00 | 1,00 |  | 0,20 | 0,20 | 0,45 | 0,15 | 0,15 | 0,10 |
| 12. | 0,45 | 0,50 | 0,45 | 1,00 | 1,00 | 1,00 |  | 0,30 | 0,25 | 0,55 | 1,00 | 0,75 | 0,85 |
| 13. | 0,30 | 0,30 | 0,30 | 0,90 | 0,70 | 0,50 |  | 0,05 | 0,00 | 0,90 | 1,00 | 1,00 | 1,00 |
| 14. | 0,40 | 0,45 | 0,35 | 1,00 | 0,80 | 0,65 |  | 0,00 | 0,00 | 0,35 | 0,35 | 1,00 | 1,00 |
| 15. | 0,15 | 0,10 | 0,10 | 0,65 | 1,00 | 0,70 |  | 0,00 | 0,00 | 0,00 | 0,40 | 1,00 | 0,80 |
| 16. | 0,55 | 0,65 | 0,20 | 1,00 | 0,75 | 0,70 |  | 0,65 | 0,65 | 0,50 | 1,00 | 1,00 | 1,00 |
| 17. | 0,35 | 0,35 | 0,20 | 0,75 | 1,00 | 0,70 |  | 0,80 | 0,35 | 0,40 | 1,00 | 0,95 | 0,75 |
| 18. | 0,35 | 0,35 | 0,15 | 1,00 | 0,70 | 0,85 |  | 0,15 | 0,15 | 0,85 | 1,00 | 1,00 | 1,00 |
| 19. | 0,35 | 0,35 | 0,15 | 0,90 | 0,80 | 0,85 |  | 0,00 | 0,15 | 0,00 | 0,50 | 0,00 | 0,00 |
| 20. | 0,20 | 0,20 | 0,25 | 0,55 | 0,00 | 0,10 |  | 0,25 | 0,20 | 0,75 | 1,00 | 0,90 | 0,90 |
| 21. | 0,10 | 0,10 | 0,05 | 0,20 | 0,00 | 0,00 |  | 0,00 | 0,00 | 0,00 | 0,20 | 1,00 | 0,90 |
| 22. | 0,35 | 0,45 | 0,25 | 0,60 | 0,70 | 0,70 |  | 0,45 | 0,50 | 0,40 | 0,80 | 0,00 | 0,00 |
| 23. | 1,00 | 1,00 | 0,25 | 1,00 | 1,00 | 1,00 |  | 0,20 | 0,15 | 0,40 | 0,05 | 0,00 | 0,00 |
| 24. | 0,35 | 0,50 | 0,05 | 0,80 | 0,25 | 0,35 |  | 0,15 | 0,15 | 0,40 | 1,00 | 1,00 | 1,00 |
| 25. | 0,15 | 0,25 | 0,50 | 1,00 | 0,75 | 0,75 |  | 0,25 | 0,10 | 0,45 | 0,00 | 0,65 | 0,45 |
| 26. | 0,35 | 0,30 | 0,30 | 0,65 | 0,40 | 0,50 |  | 0,50 | 0,60 | 0,40 | 1,00 | 0,10 | 0,05 |
| 27. | 0,10 | 0,05 | 0,05 | 0,10 | 1,00 | 1,00 |  | 0,35 | 0,40 | 0,20 | 0,35 | 0,00 | 0,00 |
| 28. | 1,00 | 1,00 | 0,20 | 1,00 | 1,00 | 1,00 |  | 0,00 | 0,00 | 0,00 | 0,00 | 0,00 | 0,00 |
| 29. | 0,20 | 0,20 | 0,20 | 0,10 | 0,00 | 0,00 |  | 0,00 | 0,00 | 0,00 | 0,00 | 0,25 | 0,20 |
| 30. | 0,25 | 0,25 | 0,15 | 1,00 | 0,90 | 0,65 |  | 0,10 | 0,10 | 0,30 | 0,10 | 0,05 | 0,05 |
| 31. | 0,00 | 0,00 | 0,00 | 1,00 | 1,00 | 1,00 |  | 0,10 | 0,10 | 0,10 | 0,10 | 0,00 | 0,00 |
| 32. | 0,30 | 0,30 | 0,20 | 0,30 | 0,00 | 0,00 |  | 0,00 | 0,00 | 0,00 | 0,00 | 0,05 | 0,00 |
| 33. | 0,00 | 0,00 | 0,35 | 0,00 | 0,00 | 0,00 |  | 0,40 | 0,35 | 0,05 | 0,15 | 0,00 | 0,00 |
| 34. | 0,00 | 0,00 | 0,00 | 0,00 | 0,00 | 0,00 |  | 0,05 | 0,10 | 0,05 | 0,25 | 0,00 | 0,00 |
| 35. | 0,00 | 0,00 | 0,00 | 0,00 | 0,00 | 0,00 |  | 0,25 | 0,20 | 0,35 | 0,20 | 0,00 | 0,00 |
| 36. | 0,05 | 0,10 | 0,05 | 0,05 | 0,00 | 0,00 |  | 0,35 | 0,25 | 0,30 | 0,35 | 0,00 | 0,00 |
| 37. | 0,00 | 0,00 | 0,00 | 0,00 | 0,00 | 0,00 |  | 0,00 | 0,00 | 0,00 | 0,00 | 0,00 | 0,00 |
| 38. | 0,05 | 0,10 | 0,05 | 0,05 | 0,00 | 0,00 |  | 0,00 | 0,00 | 0,00 | 0,00 | 0,00 | 0,00 |
| 39. | 0,00 | 0,00 | 0,00 | 0,35 | 0,05 | 0,00 |  | 0,25 | 0,10 | 0,05 | 0,00 | 0,00 | 0,00 |
| 40. | 0,15 | 0,20 | 0,05 | 0,10 | 0,00 | 0,00 |  | 0,00 | 0,00 | 0,00 | 0,00 | 0,00 | 0,00 |
| 41. | 0,00 | 0,00 | 0,00 | 0,05 | 0,05 | 0,00 |  | 0,00 | 0,00 | 0,00 | 0,00 | 0,00 | 0,00 |
| 42. | 0,25 | 0,15 | 0,10 | 0,20 | 0,00 | 0,00 |  | 0,00 | 0,00 | 0,00 | 0,00 | 0,00 | 0,00 |
| 43. | 0,00 | 0,00 | 0,00 | 0,00 | 0,00 | 0,00 |  | 0,00 | 0,00 | 0,00 | 0,00 | 0,00 | 0,00 |
| 44. | 0,10 | 0,15 | 0,00 | 0,20 | 0,00 | 0,10 |  | 0,00 | 0,00 | 0,00 | 0,00 | 0,00 | 0,00 |
| 45. | 0,00 | 0,00 | 0,00 | 0,00 | 0,00 | 0,00 |  | 0,00 | 0,00 | 0,00 | 0,00 | 0,00 | 0,00 |
| 46. | 0,15 | 0,10 | 0,00 | 0,10 | 0,00 | 0,05 |  | 0,00 | 0,00 | 0,00 | 0,00 | 0,00 | 0,00 |
| 47. | 0,00 | 0,00 | 0,00 | 0,05 | 0,00 | 0,00 |  | 0,00 | 0,00 | 0,00 | 0,00 | 0,00 | 0,00 |
| 48. | 0,00 | 0,00 | 0,00 | 0,30 | 0,00 | 0,00 |  | 0,00 | 0,00 | 0,00 | 0,00 | 0,00 | 0,00 |
| 49. | 0,00 | 0,00 | 0,00 | 0,00 | 0,00 | 0,00 |  | 0,05 | 0,05 | 0,05 | 0,05 | 0,00 | 0,00 |
| 50. | 0,00 | 0,00 | 0,00 | 0,30 | 0,00 | 0,00 |  | 0,25 | 0,20 | 0,05 | 0,10 | 0,00 | 0,00 |
| 51. | 0,00 | 0,00 | 0,00 | 0,00 | 0,00 | 0,00 |  | 0,00 | 0,00 | 0,00 | 0,00 | 0,00 | 0,00 |
| 52. | 0,00 | 0,00 | 0,00 | 0,20 | 0,05 | 0,05 |  | 0,00 | 0,00 | 0,00 | 0,00 | 0,00 | 0,00 |
| 53. | 0,00 | 0,00 | 0,00 | 0,00 | 0,00 | 0,00 |  | 0,00 | 0,00 | 0,00 | 0,00 | 0,10 | 0,00 |
| 54. | 0,00 | 0,00 | 0,00 | 0,00 | 0,00 | 0,00 |  |  |  |  |  |  |  |
| 55. | 0,00 | 0,00 | 0,00 | 0,10 | 0,00 | 0,00 |  |  |  |  |  |  |  |
| 56. | 0,00 | 0,00 | 0,00 | 0,00 | 0,00 | 0,00 |  |  |  |  |  |  |  |
